# Assessing antimicrobial resistance in pasture-based dairy farms: a 15-month surveillance study in New Zealand

**DOI:** 10.1128/aem.01390-24

**Published:** 2024-10-23

**Authors:** Rose M. Collis, Patrick J. Biggs, Sara A. Burgess, Anne C. Midwinter, Jinxin Liu, Gale Brightwell, Adrian L. Cookson

**Affiliations:** 1 Food System Integrity, AgResearch Ltd, Hopkirk Research Institute, Massey University, Palmerston North, New Zealand; 2 Molecular Epidemiology and Public Health Laboratory, School of Veterinary Science, Massey University, Palmerston North, New Zealand; 3 School of Natural Sciences, Massey University, Palmerston North, New Zealand; 4 New Zealand Food Safety Science and Research Centre, Massey University, Palmerston North, New Zealand; 5 Laboratory of Gastrointestinal Microbiology, College of Animal Science and Technology, Nanjing Agricultural University, Nanjing, China; Centers for Disease Control and Prevention, Atlanta, Georgia, USA

**Keywords:** antimicrobial resistance, shotgun metagenomic sequencing, antimicrobial resistance genes, resistome, dairy farm environment, pasture-based system, New Zealand

## Abstract

Antimicrobial resistance is a global public and animal health concern. Antimicrobial resistance genes (ARGs) have been detected in dairy farm environments globally; however, few longitudinal studies have utilized shotgun metagenomics for ARG surveillance in pasture-based systems. This 15-month study aimed to undertake a baseline survey using shotgun metagenomics to assess the relative abundance and diversity of ARGs in two pasture-based dairy farm environments in New Zealand with different management practices. There was no statistically significant difference in overall ARG relative abundance between the two dairy farms (*P* = 0.321) during the study period. Compared with overseas data, the relative abundance of ARG copies per 16S rRNA gene in feces (0.08–0.17), effluent (0.03–0.37), soil (0.20–0.63), and bulk tank milk (0.0–0.12) samples was low. Models comparing the presence or absence of resistance classes found in >10% of all feces, effluent, and soil samples demonstrated no statistically significant associations (*P* > 0.05) with “season,” and only multi-metal (*P* = 0.020) and tetracycline (*P* = 0.0003) resistance were significant at the “farm” level. Effluent samples harbored the most diverse ARGs, some with a recognized public health risk, whereas soil samples had the highest ARG relative abundance but without recognized health risks. This highlights the importance of considering the genomic context and risk of ARGs in metagenomic data sets. This study suggests that antimicrobial resistance on pasture-based dairy farms is low and provides essential baseline ARG surveillance data for such farming systems.

**IMPORTANCE:**

Antimicrobial resistance is a global threat to human and animal health. Despite the detection of antimicrobial resistance genes (ARGs) in dairy farm environments globally, longitudinal surveillance in pasture-based systems remains limited. This study assessed the relative abundance and diversity of ARGs in two New Zealand dairy farms with different management practices and provided important baseline ARG surveillance data on pasture-based dairy farms. The overall ARG relative abundance on these two farms was low, which provides further evidence for consumers of the safety of New Zealand’s export products. Effluent samples harbored the most diverse range of ARGs, some of which were classified with a recognized risk to public health, whereas soil samples had the highest ARG relative abundance; however, the soil ARGs were not classified with a recognized public health risk. This emphasizes the need to consider genomic context and risk as well as ARG relative abundance in resistome studies.

## INTRODUCTION

The development and transmission of antimicrobial resistance (AMR) is a serious global public and animal health concern. Antimicrobial resistance genes (ARGs) have been identified across numerous environments globally, including urban sewage (1–3), hospital wastewater (4), agricultural environments including dairy farms (5, 6), and soil samples from pristine environments (7). Traditional methods for AMR surveillance have focused on culture-based screening of specific bacterial pathogens such as *Escherichia coli* (8) or polymerase chain reaction (PCR) of a limited number of target ARGs (9). Advances in next-generation sequencing technologies and a reduction in their cost (10) have led to an increase in the number of studies utilizing shotgun metagenomic sequencing to study AMR. This has enabled a deeper understanding of the bacterial taxa and ARGs present in various ecosystems. An advantage of short-read shotgun metagenomic sequencing methods is the depth of sequencing data that can be achieved, allowing for the detection of genes of interest that may be present at low levels (11). Additionally, if the sequencing depth is sufficient, the genomic context of ARGs can be determined (10, 12). This contextual inference is crucial as it is important to assess (i) whether the ARGs are able to be transferred and (ii) which bacterial species harbor the resistance gene(s) to assess the public and animal health risks associated with each specific ARG (13). Acquired ARGs can be transferred via horizontal gene transfer and are of particular concern due to their ability to disseminate within bacterial populations.

The use of antimicrobials in food-producing animals has become a consumer concern (14–16), and potential transmission pathways of antimicrobial-resistant bacteria between animals, humans, and the environment have been proposed (17, 18). Globally, ARGs have been detected in the dairy farm environment including in feces, manure effluent, wastewater, and soil (5, 6, 19, 20) as well as raw bulk tank milk (21). Antimicrobial resistance is a global threat to human and animal health, driven by complex interactions across human, animal, and environmental compartments that can increase the transmission and prevalence of microorganisms with antibiotic resistance genes (22–26). For example, heavy metal (27–30) and biocide use (31) may co-select for AMR, and farm management practices such as buying in cattle (32) and feeding waste milk to calves (33, 34) may influence the prevalence and shedding of antimicrobial-resistant bacteria in the dairy farm environment. Other modifiable factors influencing AMR include seasonality, farm management practices such as the use of teat sealants, indoor housing, and antimicrobial use (17). The majority of antimicrobial usage (AMU) on New Zealand (NZ) dairy farms is for mastitis treatment and prevention (35), and therefore, AMU is likely to be higher post-lactation. Single time point sampling or collecting samples from a limited number of locations on a farm may be insufficient to accurately estimate the prevalence of antimicrobial-resistant *E. coli* (36); therefore, longitudinal study designs may be more suitable for AMR surveillance in agricultural environments, as they account for changes in AMR prevalence, seasonality, AMU, and farm management practices.

NZ dairy farm management practices differ when compared with international systems in that they are largely pasture-based (37), have a smaller average herd size of 431 cows (38), and have a low prevalence of coliform mastitis (39). The New Zealand Veterinary Association (NZVA) has announced aspirations that by 2030, antimicrobials will not be required for the maintenance of animal health and welfare (40), and instead, their use will be reserved for the treatment of disease. In NZ, prophylactic antibiotic use is only permitted with a veterinarian prescription and antibiotics of importance to human health are not permitted for growth promotion in animals (41). Compared with many international systems, NZ uses low amounts of antimicrobials in food-producing animals (42, 43) and like AMR from NZ food animals (44), the prevalence of ARGs in the NZ dairy farm environment is likely to be low. Thus, the aim of this study was to undertake a baseline survey using shotgun metagenomics to assess the relative abundance and diversity of ARGs in two pasture-based dairy farm environments in NZ.

## RESULTS

In this study, 123 metagenomic libraries were sequenced consisting of feces (*n* = 30), soil (*n* = 30), effluent (*n* = 28), milk (*n* = 24), and waste milk (*n* = 1) samples collected over a 15-month period plus controls (*n* = 10) (Table S1). Both positive and negative control DNA preparations were included in this study comprising of a mock community (Table S2; n=3), mock community log distribution (Table S3; n=3), blank reagent (*n* = 2), and PBS controls (Table S4; n=2). The shotgun metagenomic libraries were analyzed to determine their microbiome composition, relative abundance, and distribution of ARGs within the farm environment as well as identifying the bacterial hosts carrying ARGs.

Rarefaction analysis was used to determine whether the sequencing depth was sufficient to detect the resistome (defined as the collection of all resistance genes and their precursors, including those associated with both non-pathogenic and pathogenic bacteria [45]) in each sample, including resistance genes present in low relative abundance. This study used a sequencing depth of ≥40 million read pairs per sample, and rarefaction analysis suggested that this depth was sufficient to study the resistome in the effluent, feces, milk, and soil samples sequenced in this study. However, the relative abundance of ARGs was higher in three effluent samples (DF0145, DF0176, and DF0188), and the waste milk sample (DF0167) where the number of unique genes was still increasing after sub-sampling of all reads (Fig. S1), suggesting that the sequencing depth was not sufficient to detect the total resistome in these four outlier samples. The three effluent samples identified as outliers were collected from Dairy 1 from the second effluent collection point in the study. The remaining samples collected from this location (*n* = 3) had lower antimicrobial, heavy metal, and biocide resistance genes identified (< 200 genes). The waste milk sample contained a high number of ARGs, even when a low proportion of reads was sub-sampled.

### Microbial community composition of dairy farm samples

**Fig 1:**
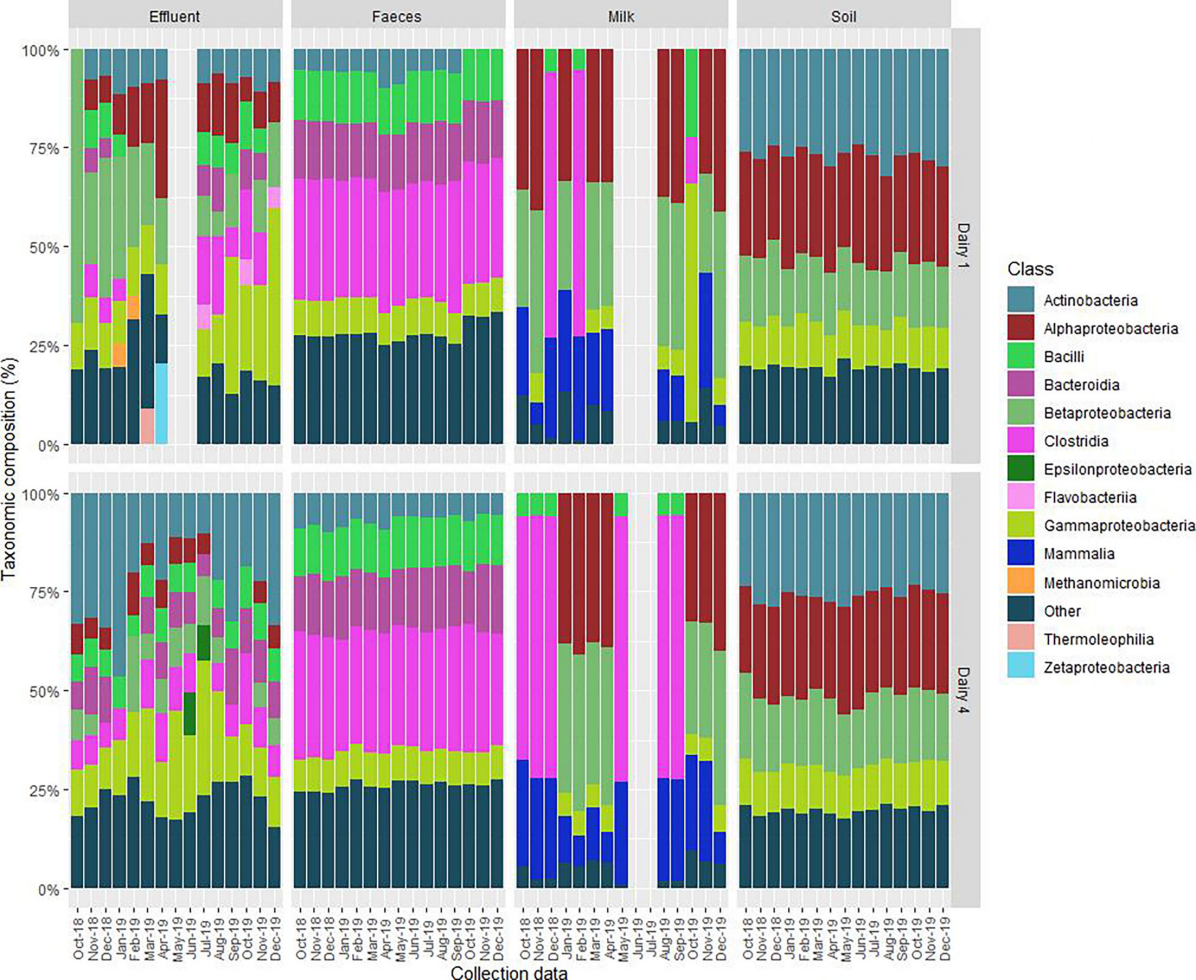
Taxonomic classification of sequencing reads at the class level from feces, farm dairy effluent, soil, and milk. Classes present in ≤5% total relative abundance were grouped together as “other.” No milk samples were collected during winter when the cows were not being milked (Dairy 1, May–July 2019; Dairy 4, June–July 2019). The October 2019 milk sample from Dairy 1 was waste milk. Effluent samples were not collected from May–June 2019 on Dairy 1.

At the taxonomic class level, the microbiome composition was similar across the fecal samples (*n* = 30) from both farms, with four predominant classes (*Clostridia*, *Bacilli*, *Gammaproteobacteria,* and *Actinobacteria*) (Fig. 1), which were also commonly detected in the effluent samples. However, the total relative abundances were more varied, and the taxonomic composition was more diverse across the different effluent samples (Fig. 1). On Dairy 1, two different collection points were used for effluent sampling, which may account for the diverse range of taxonomic classes detected within these sample types. The microbial community composition was relatively similar across the soil samples (*Actinobacteria*, *Alphaproteobacteria*, *Bacilli,* and *Gammaproteobacteria*) (Fig. 1). Two distinct microbiome profiles for the milk samples were observed: *Clostridia* followed by *Bacilli* predominated in eight samples (Dairy 1 *n* = 2; Dairy 4 *n* = 6), and *Alphaproteobacteria* and *Betaproteobacteria* in the remaining sixteen samples (Fig. 1). Samples with the latter microbiome profile had a low number of sequencing reads post-processing (106,376–335,148; Table S1) compared with the milk samples where *Clostridia* dominated (21,028,390–34,666,238; Table S1). Therefore, these contrasting microbiome profiles are likely an artifact of deep sequencing of milk samples with low DNA yields and associated with exogenous contamination from the DNA extraction kit and/or buffer solution used in sample preparation (Table S4). The waste milk sample (DF0167; collected from Dairy 1 in October 2019) had a distinct microbiome profile, which was dominated by *Gammaproteobacteria*, *Bacilli,* and *Clostridia* (Fig. 1).

**Fig 2:**
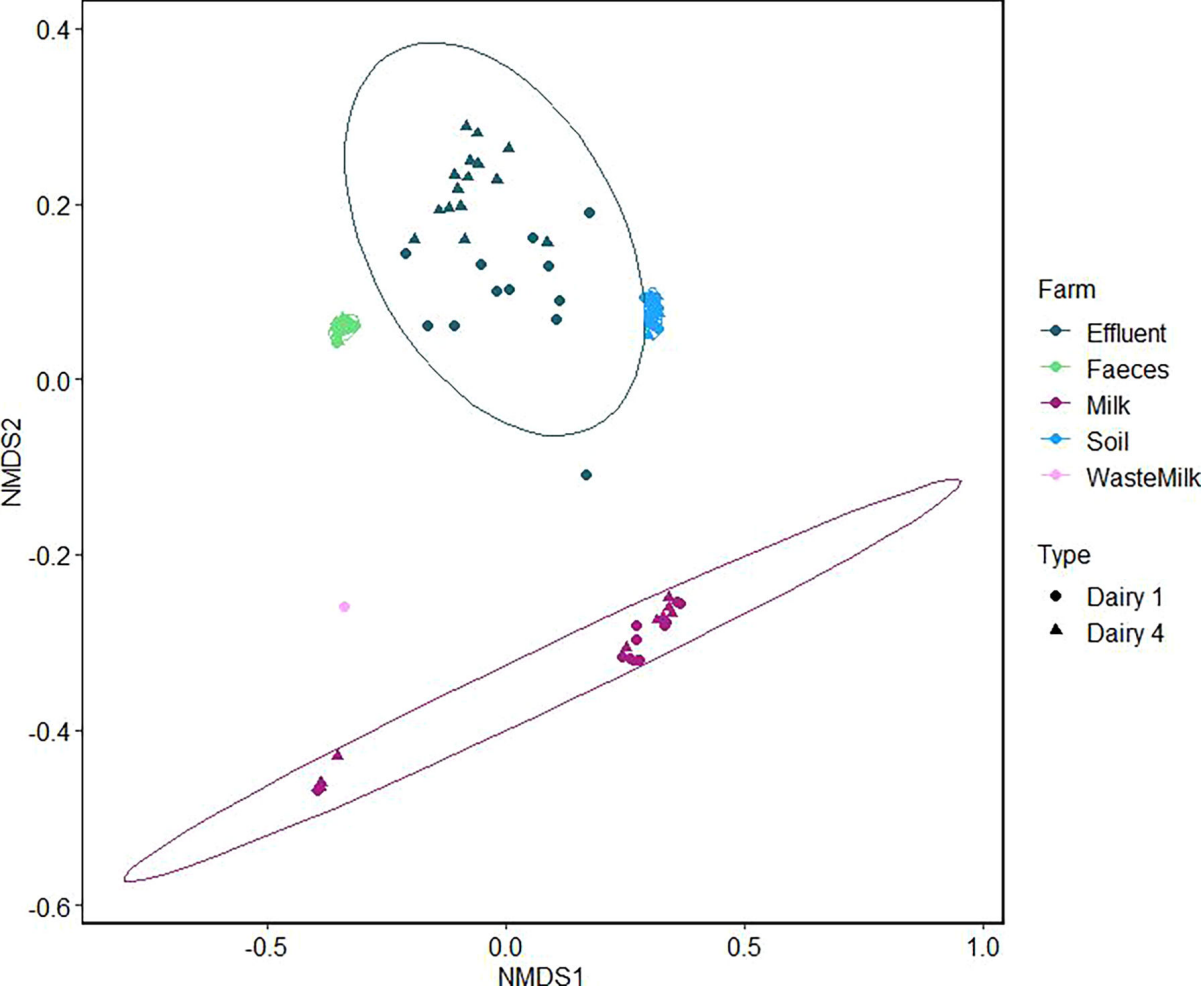
NMDS of microbiome profiles at the order level of farm samples based on Bray-Curtis dissimilarity matrix (k = 3; stress = 0.037). Samples are colored by type and shapes represent farm, as indicated in the figure key.

At the order level, the microbiome profiles clustered by sample type, irrespective of farm (Fig. 2). PERMANOVA analysis showed no statistically significant associations for both the soil and feces microbiomes (*P* > 0.05) when compared with “farm” and “season.” The fecal and soil microbiomes were homogeneous, clustering together between the farms, but milk samples clustered into two groups, independent of farm or collection date and likely associated with sequencing reads (Fig. 1). The single waste milk microbiome profile clustered separately from the other sample types. The effluent microbiome profiles were more diverse across both farms, reflecting the complex composition of effluent and different treatment methods between the two farms. PERMANOVA analysis showed both “farm” (*P* = 0.001) and “season” (*P* = 0.001) had a statistically significant effect on the effluent microbiomes. On Dairy 1, the clustering of effluent microbiome profiles was independent of the two collection sites.

### Low relative abundance of ARGs across two NZ dairy farms

**Fig 3:**
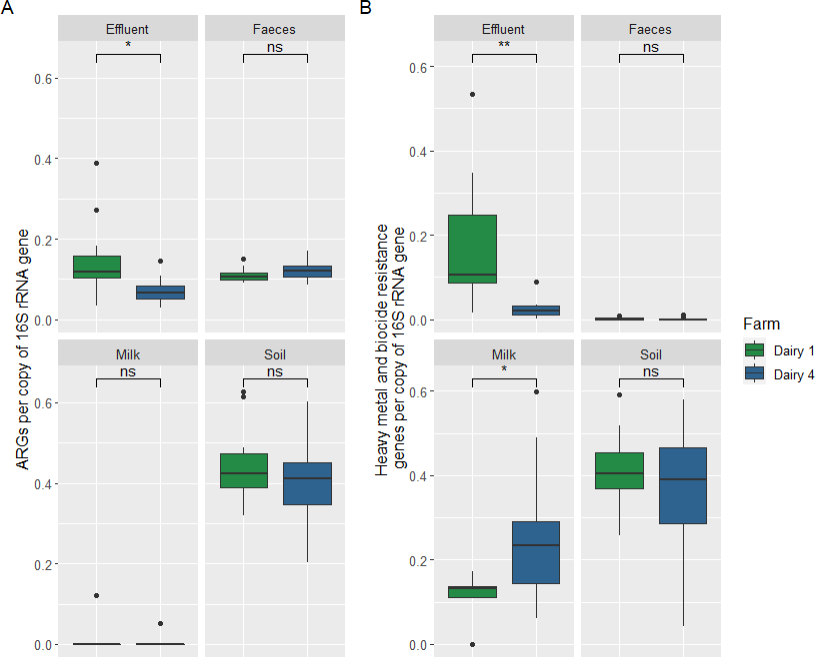
(**A**) Normalized antimicrobial resistance gene relative abundance (ARG copies per copy of 16S rRNA gene) and (**B**) heavy metal and biocide resistance gene relative abundance (heavy metal and biocide resistance genes per copy of 16S rRNA gene) in farm dairy effluent, feces, soil, and milk samples collected over a 15-month period on Dairy 1 and Dairy 4. The boxes show the median and upper and lower quartiles. The whiskers show the minimum and maximum values within the interquartile range, and the outliers are indicated by a black dot.

**Fig 4:**
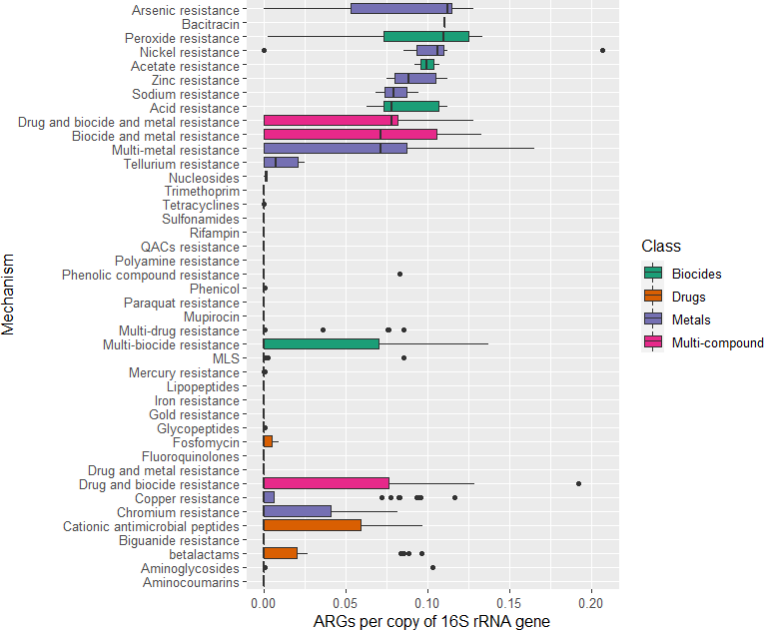
Antimicrobial, heavy metal and biocide resistance gene relative abundance (ARG copies per 16S rRNA gene) identified in the waste milk sample.

**Fig 5:**
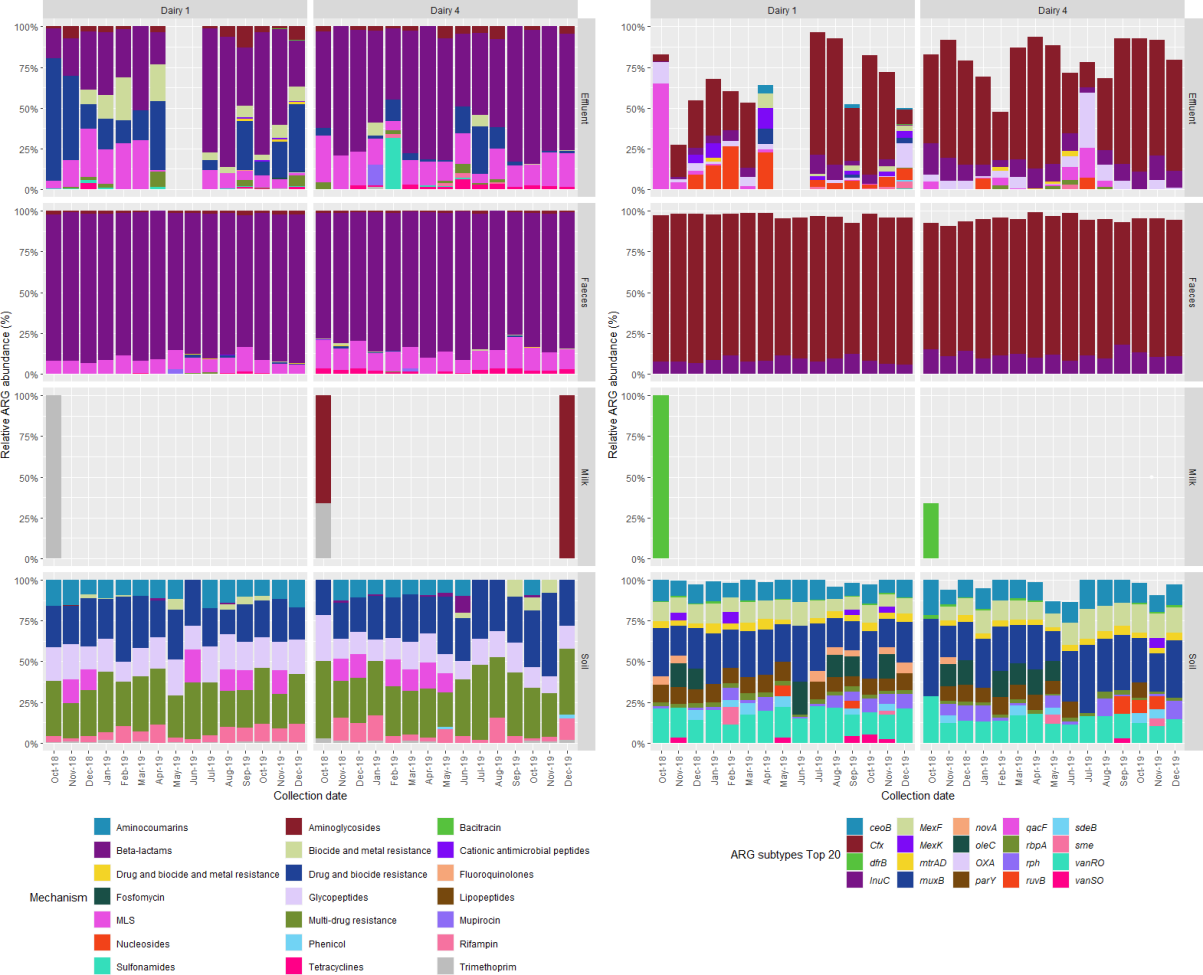
(**A**) Relative abundance of antimicrobial resistance genes and (**B**) the top 20 most abundant antimicrobial resistance genes identified from farm dairy effluent, feces, milk, and soil samples. Resistance genes are classified at the class level.

Shotgun metagenomic sequencing of feces, effluent, soil, milk, and waste milk samples from Dairy 1 and Dairy 4 identified 372 unique ARGs, representing 37 resistance classes belonging to the drugs, biocide, metal, and multi-compound classes (46). ARGs belonging to the drug and multi-compound classes (excluding biocide and heavy metal classes) ranged from 0.03 to 0.37 ARG copies per 16S rRNA gene in effluent samples, 0.08–0.17 in feces, and the lowest and highest relative abundance in milk and soil, respectively (0.0–0.12; 0.20–0.63) (Fig. 3). ARG relative abundance in soil was higher than that in effluent and feces, despite harboring fewer unique ARGs. Three effluent samples with a higher ARG relative abundance were identified as outliers (Fig. 3A), two collected from Dairy 1 in October 2018 (DF0009) and December 2019 (DF0188) and one from Dairy 4 in July 2019 (DF0115). Differences in the total ARG relative abundance (ARG copies per 16S rRNA gene) were not statistically significant between Dairy 1 and Dairy 4 over the 15-month period (*P* = 0.321). Differences in the ARG relative abundance between the effluent samples on Dairy 1 and Dairy 4 were statistically significant (*P* = 0.02), but differences in the ARG relative abundance between soil (*P* = 0.32), feces (*P* = 0.13), and milk (*P* = 0.6) samples from Dairy 1 and Dairy 4 were not statistically significant. When comparing the presence and absence of resistance classes found in >10% of all feces, effluent and soil samples at the “farm” and “season” levels, only multi-metal (*P* = 0.020) and tetracycline (*P* = 0.0003) resistance was significant at the “farm” level. There was a higher association of resistance genes in the multi-metal class on Dairy 1 and a lower odds of the tetracycline resistance class on Dairy 1 compared with Dairy 4. No statistically significant associations were found at the season level or for the other resistance classes at the farm level (*P* > 0.05). Overall, the ARG relative abundance for the singleton waste milk sample was greater than that from fecal, effluent, bulk tank milk, and soil samples (Fig. 4). Of the five sample types, effluent samples harbored the most diverse range of ARGs, with 164 unique ARGs representing 19 resistance classes, followed by feces and soil with 51 and 30 ARGs, representing 10 and 11 resistance classes, respectively (Fig. 5). The fewest unique ARGs (*n* = 2) were identified in milk, representing only two resistance classes (Fig. 5).

The ARGs detected in feces on both farms predominantly belonged to those conferring resistance to the β-lactam or macrolide, lincosamide, and streptogramin (MLS) antibiotic classes. The predominance of ARGs belonging to the β-lactam or MLS antibiotic classes is likely due to the presence of the *cfxA* and *lnuC* genes, respectively, which were identified in all fecal samples (*n* = 30; Fig. 5). The relative abundance of *cfxA* ranged from 0.07 to 0.14 ARG copies per 16S rRNA gene. *lnuC* was detected at a lower relative abundance than *cfxA* ranging from 0.01 to 0.03 ARG copies per 16S rRNA gene and encodes a lincosamide nucleotidyltransferase, which was first characterized in a clinical *Streptococcus agalactiae* on a transposon-like element (47).

The effluent samples contained the most diverse range of ARGs with resistance to antimicrobials including β-lactams, MLS, and aminoglycoside classes being predominant (Fig. 5). Resistance genes likely conferring resistance to multi-compounds were also identified (drug/biocide resistance and biocide/metal resistance). Although the ARGs identified in effluent were more diverse, they were generally detected at low relative abundance when compared with the soil resistome that had the highest ARG relative abundance. The *cfxA* gene group was identified in 27 of 28 effluent samples (0.0–0.10 ARG copies per 16S rRNA gene), and *lnuC* and *sodB,* belonging to the MLS and peroxide resistance classes, were identified in 27 of 28 and 25 of 28 effluent samples, respectively, but at a low relative abundance (0.0–0.01 and 0.0–0.06 ARG copies per 16S rRNA gene, respectively). In soil samples, identified resistance classes were comparatively similar between both farms, with multi-drug resistance, drug/biocide resistance, glycopeptides, aminocoumarins, MLS, and rifampin resistance classes (Fig. 5). ARGs were detected in a limited number of milk samples (1 of 11 on Dairy 1; 2 of 13 on Dairy 4) and belonged to the trimethoprim and aminoglycoside resistance classes, and the relative abundance of these genes was very low (0.00–0.12 and 0.00–0.05 ARG copies per 16S rRNA gene, respectively).

Differences in heavy metal and biocide resistance gene relative abundance in effluent were significant between the farms (*P* = 0.0073), with a higher relative abundance detected in Dairy 1 (0.01–0.33 ARG copies per 16S rRNA gene) when compared with Dairy 4 (0.00–0.08 ARG copies per 16S rRNA gene). For effluent samples, 149 unique heavy metal and biocide resistance genes representing 14 resistance classes were detected (Fig. S2). The *qac* gene encoding resistance to quaternary ammonium compounds, which are commonly used in disinfectants, was detected in effluent sporadically on both farms. In the milk samples, a higher relative abundance of heavy metal and biocide resistance genes was identified in Dairy 4 (0.09–0.59 ARG copies per 16S rRNA gene) when compared with Dairy 1 (0.00–0.19). Peroxide resistance (*sodB*) was detected on Dairy 4, and the remaining resistance genes all conferred mercury resistance (Fig. S2). The relative abundance of heavy metal and biocide resistance genes in feces was very low on both farms (0.00–0.01 ARG copies per 16S rRNA gene; *P* = 0.94). Heavy metal and biocide resistance gene relative abundance were higher in soil samples compared with the other sample types, and their relative abundance between soil on Dairy 1 (0.18–0.43 ARG copies per 16S rRNA gene) and Dairy 4 (0.00–0.37 ARG copies per 16S rRNA gene) did not differ (*P* = 0.39). Genes representing a diverse range of resistance classes were identified from soil, with most resistance genes conferring resistance to copper, peroxide, and iron.

Compared with all other sample types, the single waste milk sample had a higher relative abundance of antimicrobial, heavy metal, and biocide resistance genes, with 0.0–3.05 ARG copies per 16S rRNA gene (Fig. 4). From the single waste milk sample, 103 unique antimicrobial, heavy metal, or biocide resistance genes were identified, representing 14 resistance classes including copper, acid, nickel, zinc, arsenic, and peroxide resistance (Fig. S3). The most abundant resistance gene classes in the waste milk were often multi-resistant such as multi-metal (3.05 ARG copies per 16S rRNA gene), drug/biocide (2.97 ARG copies per 16S rRNA gene), biocide/metal (1.18 ARG copies per 16S rRNA gene), and multi-biocide (0.78 ARG copies per 16S rRNA gene). Drug/biocide/metal (0.70 ARG copies per 16S rRNA gene) and multi-drug (0.27 ARG copies per 16S rRNA gene) classes were also relatively abundant.

### Bacterial host range harboring contigs containing ARGs

Across the sequenced farm samples (*n* = 113), 147,822,721 contigs were assembled. Of these, 1,014 contigs (0.0007%) harbored at least one ARG and were assembled from 78.8% (88 of 113) metagenomic samples, but only 19.7% could be classified at the phylum level (200 of 1,014). The number of contigs that were taxonomically assigned reduced as the classification levels decreased down to the class (140 of 200; 70.0%), order (67 of 200; 33.5%), family (58 of 200; 29.0%), genus (42 of 200; 21.0%), and species (12 of 200; 6.0%) levels.

**Fig 6:**
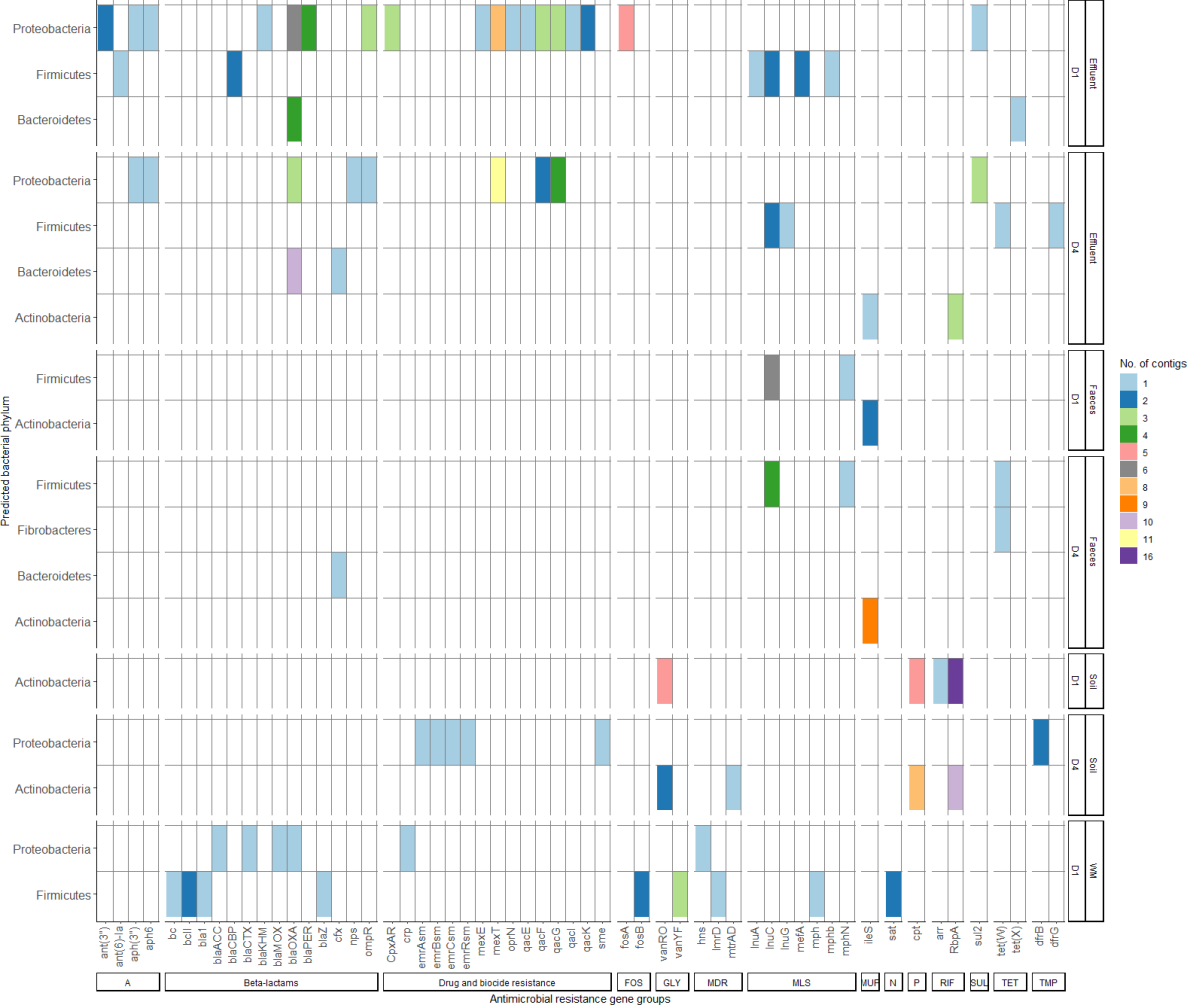
The predicted bacterial phyla of 200 contigs harboring antimicrobial resistance genes (ARGs). ARG groups were grouped per class of antibiotic and the contigs were grouped by sample type and farm. The number of contigs with the unique phyla and ARG combination is indicated by the color key on the figure legend. A, aminoglycoside; FOS, fosfomycin; GLY, glycopeptides; MDR, multi-drug resistance; MLS, macrolide, lincosamide and streptogramin; MUP, mupirocin; N, nucleosides; P, phenicol; RIF, rifampin; SUL, sulfonamides; TET, tetracycline; TMP, trimethoprim; and WM, waste milk.

Within the 200 contigs originating from 70 metagenomic samples (Fig. 6), 57 unique ARGs were identified, representing resistance to 14 antibiotic classes. Several contigs (7 of 200; 3.5%) co-harbored multiple ARGs. Interestingly, the *bla*_ACC_ (plasmid-mediated class C β-lactamase) and *crp* (repression of multidrug efflux pump expression) genes were identified on one contig assembled from the waste milk sample and were assigned to the bacterial order *Enterobacterales*. Most of the ARGs were on contigs assigned to Proteobacteria (30 genes) and Firmicutes (19 genes), followed by Actinobacteria (six genes), Bacteroidetes (three genes), and Fibrobacteres (one gene). At the phylum level, the majority of the contigs harboring ARGs were assembled from effluent samples (*n* = 102 contigs), with more contigs assembled from Dairy 1 (*n* = 57) compared with Dairy 4 (*n* = 46). ARGs identified in contigs assembled from effluent samples potentially confer resistance across 10 antimicrobial classes, with the highest number of gene groups assigned to resistance to drug and biocides (*n* = 9), β-lactams (*n* = 7), MLS (*n* = 5), and aminoglycosides (*n* = 4). These genes were predominantly found in Proteobacteria but were also detected in contigs assigned to Actinobacteria, Bacteroidetes, and Firmicutes. In addition, *qac* genes were found in contigs assigned to Proteobacteria from both farms. In total, 52 contigs harboring ARGs were assembled from soil samples with a similar number of contigs from each farm (Dairy: 1 *n* = 27; Dairy 4: *n* = 25). Fewer contigs with ARGs were assembled from fecal samples (*n* = 26), with more from Dairy 4 (*n* = 17) compared with Dairy 1 (*n* = 9). No contigs containing ARGs were assembled from milk samples. From the single waste milk sample, 19 contigs containing ARGs were assembled, with one contig co-harboring two resistance genes (*bla_ACC_* and *crp*; Table S5). The ARGs identified in the waste milk sample potentially confer resistance to β-lactams, including *bla*_CTX-M_ identified in a contig belonging to the *Enterobacterales*, drug and biocide resistance and MLS classes from contigs assigned to the phylum Proteobacteria, as well as resistance to β-lactam, fosfomycin, glycopeptide, multi-drug, MLS, and nucleoside classes detected in contigs assigned to Firmicutes.

The phylum Proteobacteria harbored the most diverse range of ARGs across the sample types. Proteobacteria was one of the predominant phyla found in the effluent samples (average: 45.1%), and the number of contigs containing ARGs classified as Proteobacteria across effluent samples from both farms was high (Dairy 1: *n* = 43; Dairy 4: *n* = 26). ARGs from contigs assigned to Proteobacteria were found in the waste milk sample (*n* = 4 contigs) and soil samples (*n* = 4 contigs), although the number of contigs was lower from these sources. Samples that had a high ARG relative abundance at the sequencing read level also had an increased number of contigs with ARGs assigned to Proteobacteria (effluent: DF0176 and DF0188; waste milk: DF0167). Despite being a predominant phylum detected in the fecal microbiome composition analysis, no Proteobacteria contigs containing ARGs were detected in fecal samples from either farm during the study.

The phylum Firmicutes also harbored a diverse range of ARGs (*n* = 19), and ARG-containing contigs were assembled from effluent (*n* = 14 contigs), feces (*n* = 13 contigs), and waste milk (*n* = 14 contigs) samples. *lnuC* was identified from all fecal samples (*n* = 30) and most effluent samples (27 of 30 samples), and 14 contigs across effluent (*n* = 4) and feces (*n* = 10) samples. Four contigs could be classified at the family level and were assigned to the *Lachnospiraceae*, *Ruminococcaceae,* and *Sporomusaceae* families (Fig. S4). ARGs were identified in contigs assigned to Bacteroidetes (*n* = 17 contigs) from effluent (*n* = 13 samples) and feces samples (*n* = 1 sample). Of these, 14 contigs harbored the *bla*_OXA_ gene, and five contigs could be classified at the family level, assigned to *Flavobacteriaceae*. One contig harbored *tet*X and was assigned to the *Sphingobacteriaceae* family. Two contigs assembled from feces and effluent (*n* = 1 each) harbored *cfx* (encodes a class A beta lactamase), and *cfxA* was identified in all fecal samples (*n* = 30) and most effluent samples (*n* = 27; 96.4%) at the sequencing read level; however, neither of these two contigs could be classified at a lower taxonomic rank.

The phylum Actinobacteria, prevalent in the soil and effluent, had a high number of contigs containing ARGs across the soil samples on both farms (Dairy 1: 13 of 15 samples; 27 contigs; Dairy 4: 11 of 15 samples; 21 contigs), as well as in feces (Dairy 1: 2 of 15, two contigs; Dairy 4: 9 of 15, nine contigs) and only effluent on Dairy 4 (4 of 15, four contigs). Effluent samples from both farms harbored the most diverse host range of contigs (Fig. 6).

## DISCUSSION

The role of the pasture-based dairy farm environment in the development and spread of AMR is not fully understood. Due to the comparatively low use of antimicrobials in food-producing animals in NZ (42–44) and the largely pasture-based dairy farm system, it was hypothesized that the relative abundance of ARGs in the dairy farm environment would be low. To test this hypothesis, shotgun metagenomics with deep-sequencing was utilized to examine and compare the resistome of environmental samples (feces, effluent, soil, and milk) collected over a 15-month period from two NZ dairy farms with contrasting farm management practices. ARG relative abundance from effluent, feces, and milk collected on Dairy 1 and Dairy 4 was relatively low. These findings suggest that pasture-based dairy farms in NZ have low levels of AMR, which provides further evidence for consumers of the safety of New Zealand’s export products. Binomial generalized linear models comparing the presence or absence of resistance classes found in >10% of all feces, effluent, and soil samples were used to test the associations of AMR with “farm” and “season,” and no statistically significant associations were observed (*P* > 0.05), except multi-metal (*P* = 0.020) and tetracycline (*P* = 0.0003) resistance were significant at the “farm” level.

Effluent harbors a diverse range of ARGs (9, 48), and applying effluent to pasture has been associated with higher detection of AmpC-producing *E. coli* on beef farms in the United Kingdom (32). A study comparing the resistome from Chinese dairy farm environments found that the farm wastewater had the most diverse ARG subtypes compared with feces and soil (49); however, soil was collected from vegetable fields fertilized by dairy manure, compared with soil from recently grazed paddocks that were analyzed in this study. The diverse number of ARGs detected in effluent may reflect the complex composition and microbiota of this sample type (50) as well as temperature (50, 51) or storage conditions (52). The effluent management strategies differed between the two farms; effluent from Dairy 4 was stored conventionally in a large pond prior to being applied to pasture, compared with Dairy 1 where no effluent was applied to pasture during the study period but instead was filtered prior to waste-water discharge using an effluent filtration system. PERMANOVA analysis of the microbiome at the order level showed that “farm” had a statistically significant effect on the effluent microbiome (*P* = 0.001), which is likely due to the different effluent management strategies between farms. “Season” also had a statistically significant (*P* = 0.001) impact on the effluent microbiome, which may have been due to variations in ambient temperature and microbial composition (such as different feed types and negligible fecal and urine inputs from the milking shed into the effluent pond during winter when cows are not milked). There was a statistically significant difference (*P* = 0.02) in the ARG relative abundance in effluent samples from the two farms, with a higher ARG relative abundance in effluent from Dairy 1 potentially caused by the contrasting effluent treatment systems or farm level factors such as the higher levels of AMU on Dairy 1 compared with Dairy 4 during this study (53). However, when modeling of the data comparing the presence or absence of resistance classes found in >10% of all feces, effluent, and soil samples was undertaken, no statistically significant associations (*P* > 0.05) with “season” were observed due to the more aggregated level of analyses undertaken.

ARG relative abundance in feces sampled in this study ranged from 0.08 to 0.17 ARG copies per 16S rRNA gene. These data are lower compared with the total ARG relative abundance in young calf feces (0.77–5.14 ARG copies per 16S rRNA gene) (54). A study comparing the fecal resistome of pre-weaned calves compared with lactating dairy cows on 17 commercial farms in the USA found a significantly higher ARG relative abundance in calves (0.43–2.9 ARG copies per 16S rRNA gene) compared with dairy cows (0.11–0.6 ARG copies per 16S rRNA gene) (55). ARGs belonging to the β-lactam resistance class were most abundant in feces due to the presence of *cfxA*, which encodes a β-lactamase and is associated with a transposon (56, 57) that may be involved in the horizontal transfer of the *cfxA* gene between *Bacteroides* and *Prevotella* species (57–59). Therefore, the high relative abundance of this gene in bovine feces may be due to Bacteroidetes being a predominant phylum detected in fecal microbiome of lactating dairy cows (60). The relative abundance of heavy metal and biocide resistance genes in feces was extremely low on both farms (0.00–0.01 resistance gene copies per 16S rRNA gene), suggesting that these genes are not prevalent in the fecal microbes from healthy dairy cattle in NZ.

The relative abundance of ARGs in milk was very low (0.00–0.12 ARG copies per 16S rRNA gene) on both farms and was only detected in three milk samples. A study comparing raw retail milk samples from across the USA found that the relative abundance of ARGs (0.0–1.0 ARG copies per 16S rRNA gene) varied from different locations and likely due to differences in the milk microbiota between samples (61). In this study, compared with ARGs, bulk tank milk samples (to be pasteurized prior to human consumption) from both Dairy 1 and Dairy 4 had a higher relative abundance of heavy metal resistance genes (0.00–0.59 resistance gene copies per 16S rRNA). All the metal resistance genes detected in milk potentially conferred mercury resistance. Other studies utilizing shotgun metagenomics to study the resistome of retail raw milk have not investigated the relative abundance of heavy metal and biocide resistance genes (21, 61). Genes conferring mercury resistance have been identified in numerous bacteria, including among members of the Firmicutes (62).

Exogenous contamination and its impact on microbiome analyses from low biomass samples such as bovine milk have been well documented (63–65). A study analyzing 16S rRNA gene sequence data from bovine milk and mammary epithelium samples found that >75% of the sequence data generated were from contaminating DNA, and for bovine milk samples, the source of this exogenous contamination was predominantly from DNA extraction kits (65). In this study, the bulk tank milk samples clustered into two distinct groups according to milk microbiome composition, which was likely an artifact of deep sequencing (40 million read pairs per sample) milk samples with low DNA yields. For these samples, the predominant genera identified were associated with exogenous contamination from the sample preparation DNA extraction kit and/or buffer solution (Table S4). Most of the genera detected in the negative controls in this study (except for *Diaphorobacter* and *Agrobacterium*) have been previously reported as contaminants of DNA extraction kits and laboratory reagents (64). ARGs were detected at a low frequency in the negative controls; however, this underscores the critical need to include such controls in studies of samples with low microbial biomass, such as bulk tank milk and anticipated low prevalence of ARGs. The presence of the two ARGs (*dfr*B and *ant*6) in bulk tank milk samples and negative controls further highlights the need for careful analysis of negative control samples in parallel with environmental samples to determine the origin of ARGs in low biomass samples. Further work including long-read sequencing is needed to elucidate the genomic context and thus significance of the ARGs detected in the bulk tank milk samples.

Feeding waste milk, which may contain low concentrations of antimicrobials (66), to young calves has been suggested as a risk factor for shedding and transmission of antimicrobial-resistant bacteria (67) and may be a source of pathogenic bacteria (66, 68). It has been suggested that feeding waste milk to calves may increase ARG relative abundance in feces (69). The waste milk disposal strategy on farm may depend upon the volume of waste milk, the criteria used to designate the milk as waste, farm management practices, as well as local regulations. Disposal of waste milk generally includes either discharge into the effluent pond (or similar storage area) or it may be fed to calves (70), which may facilitate the dissemination of antimicrobial, heavy metal, and biocide resistance genes. The inclusion of waste milk in this study was serendipitous and provided useful preliminary insights into the ARG relative abundance in waste milk on this farm. According to individual antimicrobial animal treatments recorded, five cows were receiving antimicrobial treatment for mastitis and/or between claw/footrot with products containing either β-lactam, procaine penicillin G and penethamate compounds, within 6 days prior to the sampling date. The single waste milk sample had a higher relative abundance of ARGs as well as heavy metal and biocide resistance genes (0.0–3.05 resistance gene copies per 16S rRNA gene) compared with soil, effluent, feces, and milk samples. The most abundant resistance mechanisms identified were ARGs belonging to multi-compound classes including several ARGs encoding efflux pumps or regulators, highlighting that this waste milk sample may be a source of ARGs and genes conferring resistance to other compounds such as heavy metals and biocides. However, the extent to which other waste milk samples may have been contaminated with ARGs over the course of this study is unknown. At the contig level, the detection of clinically relevant ARGs, such as *bla*_CTX-M_, which encodes an ESBL enzyme in *Enterobacterales* from waste milk, is a concern due to the potential transmission of AMR in the dairy farm environment.

Soils, including those with low anthropogenic impact, have been shown to harbor a diverse range and relative abundance of ARGs (71, 72), and many antimicrobial compounds are naturally produced by soil microorganisms (72). ARG relative abundance as well as heavy metal and biocide resistance gene relative abundance was highest in the soil samples throughout this study (0.20–0.63 ARG copies per 16S rRNA gene and 0.00–0.43 resistance gene copies per 16S rRNA gene) compared with the effluent, feces, and milk sample types. The soil microbiome composition can be highly complex (73), and both ARG and bacterial diversity were significantly correlated in a study comparing soil from three distinct ecosystems (tundra, temperate, and tropical) (71), suggesting that microbiome sample variation drives ARG diversity. In this study, soil sample microbiomes from both farms were relatively homogeneous, with the two predominant phyla being Proteobacteria and Actinobacteria, congruent with previous studies from feedlot soils (*n* = 4) in Canada (74) and soil samples collected from five US dairy farms (19). There was no statistical difference (*P* = 0.32) in ARG relative abundance in the soil samples between the two dairy farms, which is not unexpected, given the close geographical proximity of the farms (< 5 km) and the similarity in microbiome between the soil samples. ARG relative abundance in soil samples collected in this study (0.20–0.63 ARG copies per 16S rRNA gene) was comparatively low compared with soil microcosms with/without compost manure collected from cows treated with specific antibiotics (75) and ARG relative abundance in soil collected from pens housing untreated (*n* = 3) and florfenicol treated calves (*n* = 3) (76), which ranged from 0.62 to 4.53 ARG copies per 16S rRNA gene. ARG relative abundance in soil defined as pristine with little anthropogenic impact had a relative abundance of 0.05–0.28 ARG copies per 16S rRNA gene (7), which is less than the agricultural soils in this study.

The soil samples had the highest ARG relative abundance and were the third most diverse sample type (after effluent and feces), with 30 ARG groups belonging to 11 resistance classes. Although soil samples (*n* = 4) from feedlots in Canada had a smaller number of unique ARG groups compared with this study, with only nine ARG groups belonging to six classes detected (74), the sample size was considerably smaller. The ARGs from soil belonging to the multi-drug resistance class encoded multi-drug efflux pumps (*mux*B and *tap*) or regulators (*mtrAD*). Multi-drug efflux pumps are also important for functions other than AMR such as detoxification of intracellular metabolites, cell homeostasis, and bacterial virulence within plant and animal hosts (77). Several drug/biocide resistance genes were identified in soil, all of which encode efflux pumps or regulators. Despite ARGs being detected at a higher relative abundance in soil, some ARGs identified in soil have been shown to share relatively low sequence similarity to the corresponding ARG in clinical pathogens (71), although a limited number of clinical ARGs were analyzed and the phenotypic consequence of this finding is unknown.

The bacterial host and genomic context of ARGs are crucial to assess the health risk posed. In addition, some ARGs, such as efflux pumps, can have alternative functions and be involved in physiological processes unrelated to AMR. Human-associated mobile ARGs were identified as the highest risk in a recent risk framework study (13). Analysis of the ARGs used in the risk framework found that 70% (1,816 of 2,579) of ARGs were not human-associated (enriched in environments with anthropogenic impact), were classified as the lowest risk, with many not associated with mobile genetic elements (13). A diverse range of ARGs (*n* = 372) belonging to the drugs, biocide, metal, and multi-compound classes were identified in this study, many of which were identified in effluent. Despite a large number of unique ARGs being identified, the majority of these were not classified as the highest risk gene families (13). At the sequencing read level, the high-risk ARGs were *aac*6’ identified in feces; *dfr*B, from one milk sample (*dfr*B1 highest risk ARG) and *bla*_OXA_, *bla*_CTX_, and *lnu*A from effluent. Soil samples had the highest ARG relative abundance in this study; however, no high-risk ARGs were identified, suggesting that although the overall ARG relative abundance in these samples was high compared with feces, effluent, and milk, the human and animal health risk associated with these ARGs is low. The number of contigs containing ARGs that could be taxonomically classified was low, likely due to the limitations of short-read sequencing. This issue may be improved by using long-read sequencing technologies as long reads often encompass multiple genes, enhancing classification (78).

ARGs potentially conferring resistance to critically important antimicrobials in human medicine (79), including plasmid-mediated *mcr* genes conferring resistance to colistin (80), carbapenem resistance genes (81), or the resistance gene *mec*A from methicillin-resistant *S. aureus* (82), were not identified in this study. Of the ARGs detected in contigs assembled from the metagenomic samples, some high-risk ARGs were identified. Of particular concern, the *bla*_OXA_ gene was detected in a contig assigned to the *Campylobacteraceae* family from effluent, and *bla*Z and the extended-spectrum β-lactamase resistance gene *bla*_CTX-M_ were identified in *Bacillaceae* and *Enterobacterales* from waste milk, respectively. The *tet*(W) gene, which is classified as high-risk rank II gene (which includes resistance emerging in non-pathogens), was detected in *Fibrobacteraceae* and *Lachnospiraceae* from feces and effluent samples on Dairy 4, respectively. However, it is important to note that the presence of an ARG does not always correlate with a resistant phenotype. These findings suggest that although a diverse range of ARGs was detected across the feces, effluent, soil, and milk samples, albeit at a comparatively low relative abundance, the majority of the ARGs detected do not pose a high public and animal health risk. These results highlight the importance of determining the bacterial host and mobility of ARGs to assess the relevant public and animal health risk posed.

### Conclusion

ARG relative abundance in feces, effluent, soil, and bulk tank milk samples across two NZ dairy farms over a 15-month surveillance period was low compared with overseas studies. This research provides important baseline data for ARG surveillance and indicates that pasture-based systems commonly associated with NZ dairy farms may be associated with lower levels of AMR. Sustainable agricultural practices such as forage-feeding of dairy cattle can be associated with improved animal health and welfare, reduced antibiotic treatment, and AMR (17). No statistically significant difference in overall antimicrobial, heavy metal, and biocide resistance genes was observed between Dairy 1 and Dairy 4. However, differences in ARG relative abundance between effluent from the two farms were statistically significant, with a higher ARG relative abundance in effluent from Dairy 1. Effluent samples harbored the most diverse range of ARGs, some with a recognized public health risk. Soil samples had the highest ARG relative abundance (excluding the single waste milk sample); however, the ARGs in soil were not classified with a recognized risk to public or animal health, highlighting that genetic context and risk (disease-associated and mobility) as well as relative abundance should be considered when analyzing resistomes using shotgun metagenomic sequencing. However, caution should be used when comparing between studies using shotgun metagenomic sequencing methods to ensure that the ARG identification, normalization methods and sequencing depths are comparable.

## MATERIALS AND METHODS

### Study population, sample collection and processing

A comparison of farm management practices and AMU on the two farms during the study period has been previously discussed; most of the antibiotics used on Dairy 1 were penicillins, and both penicillins and first-generation cephalosporins were used on Dairy 4. Antibiotics classified as red-tier by the NZVA were infrequently used, and the sample level prevalence of EBSL-producing *E. coli* was low (53). The two dairy farms are located <5 kilometers apart, both operate a closed dairy farm system and are pasture-based systems with the use of supplementary feed when required. The farms have a spring calving system, and Dairy 1 milks once a day, whereas Dairy 4 milks twice a day. Dairy 4 also has a freestall barn (200 cow capacity). The samples were collected as described previously (53) and consisted of feces (*n* = 30), soil (*n* = 30), farm dairy effluent (*n* = 28), milk (*n* = 24), and waste milk (*n* = 1). Composite soil cores and composite cow feces from cow pats were collected from a recently grazed paddock. Farm dairy effluent is a by-product of dairy cattle being in the farm dairy, feed pads, or yards and consists of cattle feces and urine diluted with wash-down water (83). Two effluent collection sites were used on Dairy 1. For the first site, settled effluent was collected from the sump prior to filtration, but from July 2019 onward, effluent more dilute watery in composition, was collected from within a drain in the cowshed. Bulk tank milk is the collection of raw milk from multiple cows, which is stored before being collected for milk processing and would be pasteurized prior to human consumption. There are strict storage and hygiene conditions for bulk tank milk in NZ. Waste milk is any form of unsaleable milk produced on-farm. The composition of waste milk varies and may consist of (i) milk from dairy cows receiving antimicrobial treatment (either systemic or intramammary) that has a withholding period, (ii) milk from cows receiving non-antimicrobial drugs, (iii) colostrum from cows shortly after calving, and (iv) milk from ill cows or milk with a somatic cell count exceeding the saleable limit (33, 70). The milk sample collected in October 2019 from Dairy 1 was waste milk (sample DF0167), rather than bulk tank milk. This sample collection issue was discovered post-sequencing, and therefore, 24 bulk tank milk and one waste milk sample were included in the study.

For each sampling visit, feces and soil samples were homogenized and stored in 0.25 g aliquots at −80°C. To concentrate the microbial DNA and reduce the protein and fat content, approximately 400 mL of milk was centrifuged at 10,000 × *g* (Sorvall LYNX 4000 Superspeed Centrifuge, ThermoFisher Scientific, Waltham, Massachusetts, United States) for 45 min at 4°C. The fat and supernatant were discarded, and the pellet re-suspended in the remainder of the supernatant and centrifuged at 10,000 × *g* for 10 min at 4°C. The supernatant was discarded, and the pellet was washed twice in 5 mL PBS (0.01M, pH 7.3) (ThermoFisher Scientific, Waltham, Massachusetts, United States) at 10,000 × *g* for 10 min at 4°C. The re-suspended pellet was transferred and stored in duplicate at −80°C. To concentrate the effluent (based on the method described in [20]), approximately 400 mL of effluent was centrifuged at 10,000 × *g* (Sorvall LYNX 4000 Superspeed Centrifuge, ThermoFisher Scientific, Waltham, Massachusetts, United States) for 20 min at 4°C. Later, 95% of the supernatant was decanted, and the pellet was re-suspended in the remainder of the supernatant and stored at −80°C.

### DNA extraction

Genomic DNA was extracted using the Presto Stool DNA Extraction Kit (Geneaid Biotech Ltd, New Taipei City, Taiwan) according to the manufacturer’s instructions with minor modifications. Briefly, 0.25 g feces and soil and 200 µL of milk and effluent were used for the DNA extraction. For lysis, the sample was vortexed at maximum speed for 7 min (Vortex Mixer, Labnet International, New Jersey, USA) and then centrifuged at 8,000 × *g* for 2 min. Next, 500 µL of supernatant was transferred to a new tube, and 5 µL RNAse A (100 mg/mL; QIAGEN, Hilden, Germany) was added and incubated for 10 min at 37°C. To elute the DNA, 30 µL of elution buffer was added to the center of the column and left to stand for 2 min. The sample was then centrifuged, and the elution step was repeated using the buffer containing the eluted DNA and stored at −20°C. All centrifuge steps were carried out at room temperature. The DNA concentration was quantified using a Qubit 4.0 fluorometer (ThermoFisher Scientific Inc., USA) and A_260/280_ and A_260/230_ ratios were determined using the Nanodrop microvolume spectrophotometer (Nanodrop 2000c, ThermoFisher Scientific Inc., USA). DNA integrity and size were visualized on a 0.8% [wt/vol] agarose gel using a high molecular weight *Hind*III λ ladder (ThermoFisher Scientific Inc., USA).

### Shotgun metagenomic sequencing

The metagenomic sequencing run was performed on an Illumina Novaseq S4 platform with 2 × 150 paired-end reads (≥40 million paired-end reads or approximately 13 Gb per sample). The libraries were prepared using the NEBNext DNA Library Preparation Kit (New England Biolabs, Inc, Ipswich, Massachusetts, USA) and sequenced by Novogene, Singapore. The following controls (*n* = 10) were included in this study: (i) blank reagent control for each DNA extraction kit batch (*n* = 2), (ii) phosphate buffered saline (PBS, 0.01M, pH7.3) (*n* = 2) used for preparation of the milk samples, and (iii) ZymoBIOMICS Microbial Community DNA Standard (*n* = 3) (84) and ZymoBIOMICS Microbial Community DNA Standard II (log distribution) (*n* = 3) (85).

### Bioinformatic analysis

The shotgun metagenomic sequencing analysis was based on a previously described workflow (54), and the key steps of the workflow are outlined in Fig. S5. Briefly, TrimGalore v0.6.6 (86) was used for raw read trimming and quality assessment, using Cutadapt v1.18 (87) and FastQC v0.11.9 (88), respectively. A Phred quality score threshold of 20 was used. Host contamination (*Bos taurus*) and human reads (*Homo sapiens*) were removed by aligning the reads to the bovine genome UMD3.1.1 (accession number: AAFC00000000.3) and human genome GRCh38.p14 (accession number: GCF_000001405.40) respectively, using BMTagger in bmtools v3.101 (89). For the ARG normalization calculations, the number of 16S rRNA genes per sample was identified using METAXA2 v2.2 (90). The processed sequencing reads were taxonomically classified using Kraken2 v2.1.1 (91). Bacterial classifications used in this paper are those given by Kraken2 with the standard database v20200919 and do not reflect the recent name changes at the higher levels of bacterial taxonomy (92). The relative abundance of taxa within the samples was estimated at various classification levels using Bracken v2.6.0 (93) and phyla present in ≤5% total relative abundance were grouped together as “Other” for subsequent analyses. The processed reads were assembled into contigs using MEGAHIT v1.2.9 (94) with default parameters. Unless otherwise stated, all tools were used with default parameters.

### Resistance gene analysis

The resistome in the sequencing reads was analyzed following the AMR++ pipeline (https://www.meglab.org/amrplusplus/) with minor modifications. Briefly, the antimicrobial, biocide, and heavy metal resistance genes were identified by mapping the processed sequencing reads to the MEGARes database v2.0 (46) using BWA v0.7.17 with default settings (95). The SAM formatted alignment files were analyzed using ResistomeAnalyzer v2018.09.06 (https://github.com/cdeanj/resistomeanalyzer) to generate the sample resistome for each level of the database hierarchy (gene, group, mechanism, and class). For the gene-level analysis, a gene fraction (proportion of nucleotides in the reference sequence that were aligned to by at least one sequence read) threshold of 80% was used to reduce false positive hits. Rarefaction analysis was performed per sample to determine whether the sequencing depth used in this study was sufficient to detect the antimicrobial, biocide, and heavy metal resistance genes present. The SAM formatted alignment file was used as input for RarefactionAnalyzer v2018.09.06 (https://github.com/cdeanj/rarefactionanalyzer), with sub-sampling of sequencing reads at 5% increments and a gene fraction threshold of 80%. To allow for more accurate comparisons, ARG gene-level data were normalized to avoid bias associated with ARG size and the microbial load per sample and is well-utilized in this field allowing for a comparison of results from this study with other current literature. ARG relative abundance was expressed as “ARG copies per copy of 16S rRNA gene,” and normalization calculations were performed as previously described (96).

ARGs, excluding those arising from point mutations, were identified in contigs with Abricate v1.0.1 (https://github.com/tseemann/abricate) (97) using the MEGARes database v2.0 (46). Contigs containing ARGs were taxonomically classified with the Contig Annotation Tool (CAT) v5.2.1 (98) using NCBI taxonomy files and the NCBI nr database (generated 2021–01-07) with default settings.

### Statistical analysis and data visualization

Statistical analysis was performed in R v4.0.2 (99) and RStudio v1.3.959 (100). Data visualization was conducted in R using the packages “ggplot2” (101), “tidyverse” (102), and “dplyr” (103). As many of the resistance classes were detected at low levels, only antibiotic, metal, or biocide classes found in >10% of all feces, effluent, and soil samples (*n* = 88) were included, consisting of 23 out of 37 resistance classes. “glm2” (104) was used to fit binomial generalized linear models on the presence or absence of each resistance class (from the count matrix) at the sample level (response variable) to test associations with “farm” and “season” (fixed effect). The model was run separately to test the fixed effects (“farm” or “season”) with each response variable (resistance classes). For season, “emmeans” (105) was used, and the pairwise comparisons were adjusted using the Tukey method. The overall normalized relative abundance of ARGs per sample type and farm was compared using a *t*-test in “ggpubr” (106). Differences in microbiome profiles, at the taxonomic order level, based on Bray-Curtis distance measures were analyzed using non-metric multidimensional scaling (NMDS) in the “vegan” package (107). PERMANOVA analysis was performed using the adonis2 function in “vegan” (107), with the microbiome data matrix from kraken2 for each sample type (excluding milk and waste milk) as the response variable, compared with “farm” and “season” (explanatory variables) with the permutations constrained within blocks defined by “visit,” which is useful for accounting for repeated measures.

## SUPPLEMENTAL MATERIAL

Supplemental material aem.01390-24-s0001.pdf Tables S2 to S5; Figures S1 to S5.

Table S1 aem.01390-24-s0002.xlsx Sample metadata, including BioProject and BioSample accession numbers.

ASM does not own the copyrights to Supplemental Material that may be linked to, or accessed through, an article. The authors have granted ASM a non-exclusive, world-wide license to publish the Supplemental Material files. Please contact the corresponding author directly for reuse.

## References

[1] Hendriksen RS, Munk P, Njage P, van Bunnik B, McNally L, Lukjancenko O, Röder T, Nieuwenhuijse D, Pedersen SK, Kjeldgaard J, et al.. 2019. Global monitoring of antimicrobial resistance based on metagenomics analyses of urban sewage. Nat Commun 10:1124. doi:10.1038/s41467-019-08853-3 30850636 PMC6408512

[2] Rodríguez EA, Ramirez D, Balcázar JL, Jiménez JN. 2021. Metagenomic analysis of urban wastewater resistome and mobilome: a support for antimicrobial resistance surveillance in an endemic country. Environ Pollut 276:116736. doi:10.1016/j.envpol.2021.116736 33618114

[3] Munk P, Brinch C, Møller FD, Petersen TN, Hendriksen RS, Seyfarth AM, Kjeldgaard JS, Svendsen CA, van Bunnik B, Berglund F, Global Sewage Surveillance Consortium, Larsson DGJ, Koopmans M, Woolhouse M, Aarestrup FM. 2022. Genomic analysis of sewage from 101 countries reveals global landscape of antimicrobial resistance. Nat Commun 13:7251. doi:10.1038/s41467-022-34312-7 36456547 PMC9715550

[4] Marathe NP, Berglund F, Razavi M, Pal C, Dröge J, Samant S, Kristiansson E, Larsson DGJ. 2019. Sewage effluent from an Indian hospital harbors novel carbapenemases and integron-borne antibiotic resistance genes. Microbiome 7:97. doi:10.1186/s40168-019-0710-x 31248462 PMC6598227

[5] Pitta DW, Indugu N, Toth JD, Bender JS, Baker LD, Hennessy ML, Vecchiarelli B, Aceto H, Dou Z. 2020. The distribution of microbiomes and resistomes across farm environments in conventional and organic dairy herds in Pennsylvania. Environ Microbiome 15:21. doi:10.1186/s40793-020-00368-5 33902716 PMC8066844

[6] Rovira P, McAllister T, Lakin SM, Cook SR, Doster E, Noyes NR, Weinroth MD, Yang X, Parker JK, Boucher C, Booker CW, Woerner DR, Belk KE, Morley PS. 2019. Characterization of the microbial resistome in conventional and “raised without antibiotics” beef and dairy production systems. Front Microbiol 10:1980. doi:10.3389/fmicb.2019.01980 31555225 PMC6736999

[7] Li B, Chen Z, Zhang F, Liu Y, Yan T. 2020. Abundance, diversity and mobility potential of antibiotic resistance genes in pristine Tibetan Plateau soil as revealed by soil metagenomics. FEMS Microbiol Ecol 96. doi:10.1093/femsec/fiaa172 32816017

[8] Massé J, Lardé H, Fairbrother JM, Roy JP, Francoz D, Dufour S, Archambault M. 2021. Prevalence of antimicrobial resistance and characteristics of Escherichia coli isolates from fecal and manure pit samples on dairy farms in the Province of Québec, Canada. Front Vet Sci 8:654125. doi:10.3389/fvets.2021.654125 34095273 PMC8175654

[9] Guo X, Akram S, Stedtfeld R, Johnson M, Chabrelie A, Yin D, Mitchell J. 2021. Distribution of antimicrobial resistance across the overall environment of dairy farms – a case study. Sci Total Environ 788:147489. doi:10.1016/j.scitotenv.2021.147489 34134353

[10] Boolchandani M, D’Souza AW, Dantas G. 2019. Sequencing-based methods and resources to study antimicrobial resistance. Nat Rev Genet 20:356–370. doi:10.1038/s41576-019-0108-4 30886350 PMC6525649

[11] Zaheer R, Noyes N, Ortega Polo R, Cook SR, Marinier E, Van Domselaar G, Belk KE, Morley PS, McAllister TA. 2018. Impact of sequencing depth on the characterization of the microbiome and resistome. Sci Rep 8:5890. doi:10.1038/s41598-018-24280-8 29651035 PMC5897366

[12] Liu Z, Klümper U, Liu Y, Yang Y, Wei Q, Lin J-G, Gu J-D, Li M. 2019. Metagenomic and metatranscriptomic analyses reveal activity and hosts of antibiotic resistance genes in activated sludge. Environ Int 129:208–220. doi:10.1016/j.envint.2019.05.036 31129497

[13] Zhang A-N, Gaston JM, Dai CL, Zhao S, Poyet M, Groussin M, Yin X, Li L-G, van Loosdrecht MCM, Topp E, Gillings MR, Hanage WP, Tiedje JM, Moniz K, Alm EJ, Zhang T. 2021. An omics-based framework for assessing the health risk of antimicrobial resistance genes. Nat Commun 12:4765. doi:10.1038/s41467-021-25096-3 34362925 PMC8346589

[14] George A. 2018. Antimicrobial resistance, trade, food safety and security. One Health 5:6–8. doi:10.1016/j.onehlt.2017.11.004 29255786 PMC5725214

[15] Barrett JR, Innes GK, Johnson KA, Lhermie G, Ivanek R, Greiner Safi A, Lansing D. 2021. Consumer perceptions of antimicrobial use in animal husbandry: a scoping review. PLoS ONE 16:e0261010. doi:10.1371/journal.pone.0261010 34879112 PMC8654221

[16] Wemette M, Greiner Safi A, Wolverton AK, Beauvais W, Shapiro M, Moroni P, Welcome FL, Ivanek R. 2021. Public perceptions of antibiotic use on dairy farms in the United States. J Dairy Sci 104:2807–2821. doi:10.3168/jds.2019-17673 33455793

[17] Collis RM, Burgess SA, Biggs PJ, Midwinter AC, French NP, Toombs-Ruane L, Cookson AL. 2019. Extended-spectrum beta-lactamase-producing Enterobacteriaceae in dairy farm environments: A New Zealand perspective. Foodborne Pathog Dis 16:5–22. doi:10.1089/fpd.2018.2524 30418042

[18] Pattis I, Weaver L, Burgess S, Ussher JE, Dyet K. 2022. Antimicrobial resistance in New Zealand-a one health perspective. Antibiotics (Basel) 11:778. doi:10.3390/antibiotics11060778 35740184 PMC9220317

[19] Pitta DW, Dou ZX, Kumar S, Indugu N, Toth JD, Vecchiarelli B, Bhukya B. 2016. Metagenomic evidence of the prevalence and distribution patterns of antimicrobial resistance genes in dairy agroecosystems. Foodborne Pathog Dis 13:296–302. doi:10.1089/fpd.2015.2092 27046731

[20] Noyes NR, Yang X, Linke LM, Magnuson RJ, Cook SR, Zaheer R, Yang H, Woerner DR, Geornaras I, McArt JA, Gow SP, Ruiz J, Jones KL, Boucher CA, McAllister TA, Belk KE, Morley PS. 2016. Characterization of the resistome in manure, soil and wastewater from dairy and beef production systems. Sci Rep 6:24645. doi:10.1038/srep24645 27095377 PMC4837390

[21] Tóth AG, Csabai I, Krikó E, Tőzsér D, Maróti G, Patai ÁV, Makrai L, Szita G, Solymosi N. 2020. Antimicrobial resistance genes in raw milk for human consumption. Sci Rep 10:7464–7464. doi:10.1038/s41598-020-63675-4 32366826 PMC7198526

[22] Gonggrijp MA, Santman-Berends IMGA, Heuvelink AE, Buter GJ, van Schaik G, Hage JJ, Lam TJGM. 2016. Prevalence and risk factors for extended-spectrum β-lactamase- and AmpC-producing Escherichia coli in dairy farms. J Dairy Sci 99:9001–9013. doi:10.3168/jds.2016-11134 27638264

[23] Ferroni L, Albini E, Lovito C, Blasi F, Maresca C, Massacci FR, Orsini S, Tofani S, Pezzotti G, Diaz Vicuna E, Forte C, Magistrali CF. 2022. Antibiotic consumption is a major driver of antibiotic resistance in calves raised on Italian cow-calf beef farms. Res Vet Sci 145:71–81. doi:10.1016/j.rvsc.2022.01.010 35176652

[24] Laxminarayan R, Duse A, Wattal C, Zaidi AKM, Wertheim HFL, Sumpradit N, Vlieghe E, Hara GL, Gould IM, Goossens H, et al.. 2013. Antibiotic resistance-the need for global solutions. Lancet Infect Dis 13:1057–1098. doi:10.1016/S1473-3099(13)70318-9 24252483

[25] Meijs AP, Rozwandowicz M, Hengeveld PD, Dierikx CM, de Greeff SC, van Duijkeren E, van Dissel JT. 2024. Human carriage of ESBL/pAmpC-producing Escherichia coli and Klebsiella pneumoniae in relation to the consumption of raw or undercooked vegetables, fruits, and fresh herbs. Microbiol Spectr 12:e0284923. doi:10.1128/spectrum.02849-23 38206033 PMC10845978

[26] Benbow A, Clarke M, Yates C, Montgomery R, Staniforth K, Boswell T, Prescott K, Mahida N. 2024. Hospital-wide healthcare-associated carbapenemase-producing Enterobacterales outbreak: risks of electric floor scrubbers in catering facilities and kitchens. J Hosp Infect 146:59–65. doi:10.1016/j.jhin.2024.01.016 38341149

[27] Knapp CW, McCluskey SM, Singh BK, Campbell CD, Hudson G, Graham DW. 2011. Antibiotic resistance gene abundances correlate with metal and geochemical conditions in archived Scottish soils. PLoS One 6:e27300. doi:10.1371/journal.pone.0027300 22096547 PMC3212566

[28] Ji XL, Shen QH, Liu F, Ma J, Xu G, Wang YL, Wu MH. 2012. Antibiotic resistance gene abundances associated with antibiotics and heavy metals in animal manures and agricultural soils adjacent to feedlots in Shanghai; China. J Hazard Mater 235–236:178–185. doi:10.1016/j.jhazmat.2012.07.040 22868748

[29] Thomas JC, Oladeinde A, Kieran TJ, Finger JW, Bayona-Vásquez NJ, Cartee JC, Beasley JC, Seaman JC, McArthur JV, Rhodes OE, Glenn TC. 2020. Co-occurrence of antibiotic, biocide, and heavy metal resistance genes in bacteria from metal and radionuclide contaminated soils at the Savannah River Site. Microb Biotechnol 13:1179–1200. doi:10.1111/1751-7915.13578 32363769 PMC7264878

[30] Chen J, Li J, Zhang H, Shi W, Liu Y. 2019. Bacterial heavy-metal and antibiotic resistance genes in a copper tailing dam area in northern China. Front Microbiol 10:1916–1916. doi:10.3389/fmicb.2019.01916 31481945 PMC6710345

[31] Romero JL, Grande Burgos MJ, Pérez-Pulido R, Gálvez A, Lucas R. 2017. Resistance to antibiotics, biocides, preservatives and metals in bacteria isolated from seafoods: co-selection of strains resistant or tolerant to different classes of compounds. Front Microbiol 8:1650. doi:10.3389/fmicb.2017.01650 28912764 PMC5583239

[32] Velasova M, Smith RP, Lemma F, Horton RA, Duggett NA, Evans J, Tongue SC, Anjum MF, Randall LP. 2019. Detection of extended-spectrum β-lactam, AmpC and carbapenem resistance in Enterobacteriaceae in beef cattle in Great Britain in 2015. J Appl Microbiol 126:1081–1095. doi:10.1111/jam.14211 30693606

[33] Aust V, Knappstein K, Kunz HJ, Kaspar H, Wallmann J, Kaske M. 2013. Feeding untreated and pasteurized waste milk and bulk milk to calves: effects on calf performance, health status and antibiotic resistance of faecal bacteria. J Anim Physiol Anim Nutr (Berl) 97:1091–1103. doi:10.1111/jpn.12019 23205592

[34] Brunton LA, Reeves HE, Snow LC, Jones JR. 2014. A longitudinal field trial assesing the impact of feeding waste milk containing antibiotic residues on the prevalence of ESBL-producing Escherichia coli in calves. Prev Vet Med 117:403–412. doi:10.1016/j.prevetmed.2014.08.005 25172121

[35] Compton C, McDougall S. 2014. Patterns of antibiotic sales to Waikato dairy farms. Vetscript 27:22–24.

[36] Schubert H, Morley K, Puddy EF, Arbon R, Findlay J, Mounsey O, Gould VC, Vass L, Evans M, Rees GM, Barrett DC, Turner KM, Cogan TA, Avison MB, Reyher KK. 2021. Reduced antibacterial drug resistance and bla_CTX-M_ β-Lactamase gene carriage in cattle-associated Escherichia coli at low temperatures, at sites dominated by older animals, and on pastureland: implications for surveillance. Appl Environ Microbiol 87:e01468-20. doi:10.1128/AEM.01468-20 PMC8105006 33397699

[37] Holmes CW, Brookes IM, Garrick DJ, Mackenzie DDS, Parkinson TJ, Wilson GF. 2002. Pastoral dairy farming in New Zealand. Massey University.

[38] Dairy NZ Limited. 2019. QuickStats about dairying. New Zealand

[39] Lacy-Hulbert J, Williamson J, Kolver E, Doohan H, Shelgren J. n.d. Is coliform mastitis an emerging issue? In . New Zealand Veterinary Association.

[40] Anonymous. 2016. 2016 New Zealand organic market report. Organics Aotearoa New Zealand. Auckland, New Zealand

[41] Anonymous. 1997. Agricultural compounds and veterinary medicines act 1997. Wellington, New Zealand. Available from: http://www.legislation.govt.nz/act/public/1997/0087/latest/DLM414577.html

[42] Hillerton JE, Bryan MA, Beattie BH, Scott D, Millar A, French NP. 2021. Use of antimicrobials for food animals in New Zealand: updated estimates to identify a baseline to measure targeted reductions. N Z Vet J 69:180–185. doi:10.1080/00480169.2021.1890648 33720815

[43] Hillerton JE, Irvine CR, Bryan MA, Scott D, Merchant SC. 2017. Use of antimicrobials for animals in New Zealand, and in comparison with other countries. N Z Vet J 65:71–77. doi:10.1080/00480169.2016.1171736 27030313

[44] Cornelius AJ, Carr SD, Bakker SN, Haysom IW, Dyet KH. 2024. Antimicrobial resistance in selected bacteria from food animals in New Zealand 2018-2022. J Food Prot 87:100245. doi:10.1016/j.jfp.2024.100245 38387832

[45] Wright GD. 2007. The antibiotic resistome: the nexus of chemical and genetic diversity. Nat Rev Microbiol 5:175–186. doi:10.1038/nrmicro1614 17277795

[46] Doster E, Lakin SM, Dean CJ, Wolfe C, Young JG, Boucher C, Belk KE, Noyes NR, Morley PS. 2020. MEGARes 2.0: a database for classification of antimicrobial drug, biocide and metal resistance determinants in metagenomic sequence data. Nucleic Acids Res 48:D561–D569. doi:10.1093/nar/gkz1010 31722416 PMC7145535

[47] Achard A, Villers C, Pichereau V, Leclercq R. 2005. New lnu(C) gene conferring resistance to lincomycin by nucleotidylation in Streptococcus agalactiae UCN36. Antimicrob Agents Chemother 49:2716–2719. doi:10.1128/AAC.49.7.2716-2719.2005 15980341 PMC1168647

[48] Checcucci A, Trevisi P, Luise D, Modesto M, Blasioli S, Braschi I, Mattarelli P. 2020. Exploring the animal waste resistome: the spread of antimicrobial resistance genes through the use of livestock manure. Front Microbiol 11:1416. doi:10.3389/fmicb.2020.01416 32793126 PMC7387501

[49] Kang J, Liu Y, Chen X, Xu F, Wang H, Xiong W, Li X. 2022. Metagenomic insights into the antibiotic resistomes of typical Chinese dairy farm environments. Front Microbiol 13:990272. doi:10.3389/fmicb.2022.990272 36246251 PMC9555277

[50] Qian X, Sun W, Gu J, Wang X-J, Sun J-J, Yin Y-N, Duan M-L. 2016. Variable effects of oxytetracycline on antibiotic resistance gene abundance and the bacterial community during aerobic composting of cow manure. J Hazard Mater 315:61–69. doi:10.1016/j.jhazmat.2016.05.002 27179201

[51] Xie W-Y, Yang X-P, Li Q, Wu L-H, Shen Q-R, Zhao F-J. 2016. Changes in antibiotic concentrations and antibiotic resistome during commercial composting of animal manures. Environ Pollut 219:182–190. doi:10.1016/j.envpol.2016.10.044 27814534

[52] Sukhum KV, Vargas RC, Boolchandani M, D’Souza AW, Patel S, Kesaraju A, Walljasper G, Hegde H, Ye Z, Valenzuela RK, Gunderson P, Bendixsen C, Dantas G, Shukla SK. 2021. Manure microbial communities and resistance profiles reconfigure after transition to manure pits and differ from those in fertilized field soil. MBio 12:e00798-21. doi:10.1128/mBio.00798-21 33975936 PMC8262906

[53] Collis RM, Biggs PJ, Burgess SA, Midwinter AC, Brightwell G, Cookson AL. 2022. Prevalence and distribution of extended-spectrum β-lactamase and AmpC-producing Escherichia coli in two New Zealand dairy farm environments. Front Microbiol 13:960748. doi:10.3389/fmicb.2022.960748 36033848 PMC9403332

[54] Liu J, Taft DH, Maldonado-Gomez MX, Johnson D, Treiber ML, Lemay DG, DePeters EJ, Mills DA. 2019. The fecal resistome of dairy cattle is associated with diet during nursing. Nat Commun 10:4406. doi:10.1038/s41467-019-12111-x 31562300 PMC6765000

[55] Haley BJ, Kim S-W, Salaheen S, Hovingh E, Van Kessel JAS. 2020. Differences in the microbial community and resistome structures of feces from preweaned calves and lactating dairy cows in commercial dairy herds. Foodborne Pathog Dis 17:494–503. doi:10.1089/fpd.2019.2768 32176535

[56] Smith CJ, Tribble GD, Bayley DP. 1998. Genetic elements of bacteroides species: a moving story. Plasmid 40:12–29. doi:10.1006/plas.1998.1347 9657930

[57] García N, Gutiérrez G, Lorenzo M, García JE, Píriz S, Quesada A. 2008. Genetic determinants for cfxA expression in Bacteroides strains isolated from human infections. J Antimicrob Chemother 62:942–947. doi:10.1093/jac/dkn347 18775891

[58] Ferreira LQ, Avelar KES, Vieira JMBD, de Paula GR, Colombo APV, Domingues RMCP, Ferreira MCS. 2007. Association between the cfxA gene and transposon Tn4555 in bacteroides distasonis strains and other bacteroides species. Curr Microbiol 54:348–353. doi:10.1007/s00284-006-0411-0 17486409

[59] Giraud-Morin C, Madinier I, Fosse T. 2003. Sequence analysis of cfxA2-like beta-lactamases in Prevotella species. J Antimicrob Chemother 51:1293–1296. doi:10.1093/jac/dkg221 12697645

[60] Hagey JV, Bhatnagar S, Heguy JM, Karle BM, Price PL, Meyer D, Maga EA. 2019. Fecal microbial communities in a large representative cohort of California dairy cows. Front Microbiol 10:1093. doi:10.3389/fmicb.2019.01093 31156599 PMC6532609

[61] Liu J, Zhu Y, Jay-Russell M, Lemay DG, Mills DA. 2020. Reservoirs of antimicrobial resistance genes in retail raw milk. Microbiome 8:99. doi:10.1186/s40168-020-00861-6 32591006 PMC7320593

[62] Boyd ES, Barkay T. 2012. The mercury resistance operon: from an origin in a geothermal environment to an efficient detoxification machine. Front Microbiol 3:349. doi:10.3389/fmicb.2012.00349 23087676 PMC3466566

[63] Pollock J, Salter SJ, Nixon R, Hutchings MR. 2021. Milk microbiome in dairy cattle and the challenges of low microbial biomass and exogenous contamination. Anim Microbiome 3:80. doi:10.1186/s42523-021-00144-x 34794515 PMC8600933

[64] Salter SJ, Cox MJ, Turek EM, Calus ST, Cookson WO, Moffatt MF, Turner P, Parkhill J, Loman NJ, Walker AW. 2014. Reagent and laboratory contamination can critically impact sequence-based microbiome analyses. BMC Biol 12:87. doi:10.1186/s12915-014-0087-z 25387460 PMC4228153

[65] Dean CJ, Deng Y, Wehri TC, Pena-Mosca F, Ray T, Crooker BA, Godden SM, Caixeta LS, Noyes NR. 2023. The impact of kit, environment and sampling contamination on the observed microbiome of bovine milk. bioRxiv. doi:10.1101/2023.11.07.566052 PMC11237780 38785438

[66] Tempini PN, Aly SS, Karle BM, Pereira RV. 2018. Multidrug residues and antimicrobial resistance patterns in waste milk from dairy farms in central California. J Dairy Sci 101:8110–8122. doi:10.3168/jds.2018-14398 30126599

[67] Duse A, Waller KP, Emanuelson U, Unnerstad HE, Persson Y, Bengtsson B. 2015. Risk factors for antimicrobial resistance in fecal Escherichia coli from preweaned dairy calves. J Dairy Sci 98:500–516. doi:10.3168/jds.2014-8432 25465547

[68] Edrington TS, Garcia Buitrago JA, Hagevoort GR, Loneragan GH, Bricta-Harhay DM, Callaway TR, Anderson RC, Nisbet DJ. 2018. Effect of waste milk pasteurization on fecal shedding of Salmonella in preweaned calves. J Dairy Sci 101:9266–9274. doi:10.3168/jds.2018-14668 30077443

[69] Thames CH, Pruden A, James RE, Ray PP, Knowlton KF. 2012. Excretion of antibiotic resistance genes by dairy calves fed milk replacers with varying doses of antibiotics. Front Microbio 3:139–139. doi:10.3389/fmicb.2012.00139 PMC3322659 22514550

[70] Ricci A, Allende A, Bolton D, Chemaly M, Davies R, Fernández Escámez PS, Girones R, Koutsoumanis K, Lindqvist R, Nørrung B, et al.. 2017. Risk for the development of Antimicrobial Resistance (AMR) due to feeding of calves with milk containing residues of antibiotics. EFS2 15:e04665–e04665. doi:10.2903/j.efsa.2017.4665 PMC7372110 32704309

[71] Qian X, Gunturu S, Guo J, Chai B, Cole JR, Gu J, Tiedje JM. 2021. Metagenomic analysis reveals the shared and distinct features of the soil resistome across tundra, temperate prairie, and tropical ecosystems. Microbiome 9:108–108. doi:10.1186/s40168-021-01047-4 33990222 PMC8122544

[72] Cytryn E. 2013. The soil resistome: the anthropogenic, the native, and the unknown. Soil Biol Biochem 63:18–23. doi:10.1016/j.soilbio.2013.03.017

[73] Fierer N. 2017. Embracing the unknown: disentangling the complexities of the soil microbiome. Nat Rev Microbiol 15:579–590. doi:10.1038/nrmicro.2017.87 28824177

[74] Zaheer R, Lakin SM, Polo RO, Cook SR, Larney FJ, Morley PS, Booker CW, Hannon SJ, Van Domselaar G, Read RR, McAllister TA. 2019. Comparative diversity of microbiomes and resistome in beef feedlots, downstream environments and urban sewage influent. BMC Microbiol 19:197–197. doi:10.1186/s12866-019-1548-x 31455230 PMC6712873

[75] Chen C, Pankow CA, Oh M, Heath LS, Zhang L, Du P, Xia K, Pruden A. 2019. Effect of antibiotic use and composting on antibiotic resistance gene abundance and resistome risks of soils receiving manure-derived amendments. Environ Int 128:233–243. doi:10.1016/j.envint.2019.04.043 31059918

[76] Liu J, Yu F, Call DR, Mills DA, Zhang A, Zhao Z. 2021. On-farm soil resistome is modified after treating dairy calves with the antibiotic florfenicol. Sci Total Environ 750:141694. doi:10.1016/j.scitotenv.2020.141694 32871373

[77] Martinez JL, Sánchez MB, Martínez-Solano L, Hernandez A, Garmendia L, Fajardo A, Alvarez-Ortega C. 2009. Functional role of bacterial multidrug efflux pumps in microbial natural ecosystems. FEMS Microbiol Rev 33:430–449. doi:10.1111/j.1574-6976.2008.00157.x 19207745

[78] Portik DM, Brown CT, Pierce-Ward NT. 2022. Evaluation of taxonomic classification and profiling methods for long-read shotgun metagenomic sequencing datasets. BMC Bioinformatics 23:541. doi:10.1186/s12859-022-05103-0 36513983 PMC9749362

[79] Collignon PC, Conly JM, Andremont A, McEwen SA, Aidara-Kane A, World Health Organization Advisory Group. 2016. World Health Organization ranking of antimicrobials according to their importance in human medicine: a critical step for developing risk management strategies to control antimicrobial resistance from food animal production. Clin Infect Dis 63:1087–1093. doi:10.1093/cid/ciw475 27439526

[80] Liu YY, Wang Y, Walsh TR, Yi LX, Zhang R, Spencer J, Doi Y, Tian GB, Dong BL, Huang XH, Yu LF, Gu DX, Ren HW, Chen XJ, Lv LC, He DD, Zhou HW, Liang ZS, Liu JH, Shen JZ. 2016. Emergence of plasmid-mediated colistin resistance mechanism MCR-1 in animals and human beings in China: a microbiological and molecular biological study. Lancet Infect Dis 16:161–168. doi:10.1016/S1473-3099(15)00424-7 26603172

[81] Codjoe FS, Donkor ES. 2017. Carbapenem resistance: a review. Med Sci (Basel) 6:1–1. doi:10.3390/medsci6010001 29267233 PMC5872158

[82] Hiramatsu K, Ito T, Tsubakishita S, Sasaki T, Takeuchi F, Morimoto Y, Katayama Y, Matsuo M, Kuwahara-Arai K, Hishinuma T, Baba T. 2013. Genomic basis for methicillin resistance in Staphylococcus aureus. Infect Chemother 45:117–136. doi:10.3947/ic.2013.45.2.117 24265961 PMC3780952

[83] Houlbrooke DJ, Horne DJ, Hedley MJ, Hanly JA, Snow VO. 2004. A review of literature on the land treatment of farm‐dairy effluent in New Zealand and its impact on water quality. N Z J Agric Res 47:499–511. doi:10.1080/00288233.2004.9513617

[84] Zymo Research Corp. 2024 ZymoBIOMIC microbial community DNA standard. Available from: https://files.zymoresearch.com/protocols/_d6305_d6306_zymobiomics_microbial_community_dna_standard.pdf

[85] Zymo Research Corp. 2024 ZymoBIOMIC microbial community DNA standard ii (log distribution). Available from: https://files.zymoresearch.com/protocols/_d6311_zymobiomics_microbial_community_dna_standard_ii_(log_distribution).pdf

[86] Krueger F. 2019. TrimGalore. Available from: https://github.com/FelixKrueger/TrimGalore

[87] Martin M. 2011. Cutadapt removes adapter sequences from high-throughput sequencing reads. EMBnet j 17:10. doi:10.14806/ej.17.1.200

[88] Andrews S. 2010. FastQC:a quality control tool for high throughput sequence data. Available from: http://www.bioinformatics.babraham.ac.uk/projects/fastqc/

[89] Rotmistrovsky K, Agarwala R. 2011. BMTagger: best match tagger for removing human reads from metagenomics datasets. Available from: ftp://ftp.ncbi.nlm.nih.gov/pub/agarwala/bmtagger/

[90] Bengtsson-Palme J, Hartmann M, Eriksson KM, Pal C, Thorell K, Larsson DGJ, Nilsson RH. 2015. METAXA2: improved identification and taxonomic classification of small and large subunit rRNA in metagenomic data. Mol Ecol Resour 15:1403–1414. doi:10.1111/1755-0998.12399 25732605

[91] Wood DE, Lu J, Langmead B. 2019. Improved metagenomic analysis with Kraken 2. Genome Biol 20:257. doi:10.1186/s13059-019-1891-0 31779668 PMC6883579

[92] Oren A, Garrity GM. 2021. Valid publication of the names of forty-two phyla of prokaryotes. Int J Syst Evol Microbiol 71. doi:10.1099/ijsem.0.005056 34694987

[93] Lu J, Breitwieser FP, Thielen P, Salzberg SL. 2017. Bracken: estimating species abundance in metagenomics data. PeerJ Comput Sci 3:e104. doi:10.7717/peerj-cs.104 PMC12016282 40271438

[94] Li D, Liu CM, Luo R, Sadakane K, Lam TW. 2015. MEGAHIT: an ultra-fast single-node solution for large and complex metagenomics assembly via succinct de Bruijn graph. Bioinformatics 31:1674–1676. doi:10.1093/bioinformatics/btv033 25609793

[95] Li H, Durbin R. 2009. Fast and accurate short read alignment with burrows-wheeler transform. Bioinformatics 25:1754–1760. doi:10.1093/bioinformatics/btp324 19451168 PMC2705234

[96] Li B, Yang Y, Ma LP, Ju F, Guo F, Tiedje JM, Zhang T. 2015. Metagenomic and network analysis reveal wide distribution and co-occurrence of environmental antibiotic resistance genes. ISME J 9:2490–2502. doi:10.1038/ismej.2015.59 25918831 PMC4611512

[97] Seemann T. 2017. Abricate. Github. Available from: https://github.com/tseemann/abricate

[98] von Meijenfeldt FAB, Arkhipova K, Cambuy DD, Coutinho FH, Dutilh BE. 2019. Robust taxonomic classification of uncharted microbial sequences and bins with CAT and BAT. Genome Biol 20:217–217. doi:10.1186/s13059-019-1817-x 31640809 PMC6805573

[99] R Core Team. 2020. R: a language and environment for statistical computing. R Foundation for Statistical Computing, Vienna, Austria. Available from: https://www.r-project.org/

[100] R Core Team. 2019. RStudio: integrated development for R. RStudio, Inc., Boston, MA. Available from: http://www.rstudio.com/

[101] Wickham H. 2016. ggplot2: elegant graphics for data analysis. Springer-Verlag, New York. Available from: https://ggplot2.tidyverse.org

[102] Wickham Hadley, Averick M, Bryan J, Chang W, McGowan L, François R, Grolemund G, Hayes A, Henry L, Hester J, Kuhn M, Pedersen T, Miller E, Bache S, Müller K, Ooms J, Robinson D, Seidel D, Spinu V, Takahashi K, Vaughan D, Wilke C, Woo K, Yutani H. 2019. Welcome to the tidyverse. JOSS 4:1686. doi:10.21105/joss.01686

[103] Wickham H, Romain F, Lionel H, Müller K. 2021. dplyr: a grammar of data manipulation, https://cran.r-project.org/package=dplyr.

[104] Marschner IC. 2011. glm2: fitting generalized linear models with convergence problems. R J 3:12. doi:10.32614/RJ-2011-012

[105] Russell VL. 2021. Emmeans: estimated marginal Means, aka least-squares means. Available from: https://cran.r-project.org/package=emmeans

[106] Kassambara A. 2016. V0.4.0. Package “Ggpubr.” Available from: https://rpkgs.datanovia.com/ggpubr/index.html

[107] Oksanen J, Guillaume Blanchet F, Friendly M, Kindt R, Legendre P, McGlinn D, Minchin PR, O’Hara RB, Simpson GL, Solymos P, Stevens MHH, Szoecs E, Wagner H. 2020. Vegan: community ecology package. Available from: https://cran.r-project.org/package=vegan

