# Supplementary material for: Assessing antimicrobial resistance in pasture-based dairy farms: a 15-month surveillance study in New Zealand
Source: Appl Environ Microbiol. 2024 Oct 23;90(11):e01390-24. doi: 10.1128/aem.01390-24 (PMC11577782; doi:10.1128/aem.01390-24)
Supplement: Supplemental material — Tables S2 to S5; Figures S1 to S5. [file aem.01390-24-s0001.pdf]

Supplemental Materials

Table S2: The expected and actual proportion of reads taxonomically classified in the DNA mock community sequencing control

**Species**	**Expected (%)**	**Reads (%)**
Pseudomonas aeruginosa	12	17.1
Escherichia coli	12	11.2
Salmonella enterica	12	17.2
Bacillus subtilis	12	6.6
Lactobacillus fermentum	12	9.1
Enterococcus faecalis	12	10.0
Listeria monocytogenes	12	10.2
Staphylococcus aureus	12	9.8
Saccharomyces cerevisiae	2	Not assessed
Cryptococcus neoformans	2	Not assessed

Table S3: Genomic DNA content and the proportion of reads taxonomically classified in the microbial community log distribution standard

**Species**	**Expected (%)**	**Reads (%)**
Listeria monocytogenes	89.1	85.4
Pseudomonas aeruginosa	8.9	12.6
Bacillus subtilis	0.89	0.4
Saccharomyces cerevisiae	0.89	Not assessed
Escherichia coli	0.089	0.1
Salmonella enterica	0.089	0.1
Lactobacillus fermentum	0.0089	0
Enterococcus faecalis	0.00089	0.04
Cryptococcus neoformans	0.00089	Not assessed
Staphylococcus aureus	0.000089	0

Table S4: The expected and actual proportion of reads taxonomically classified in the DNA mock community sequencing control

**Species**	**Expected (%)**	**Reads (%)**
Pseudomonas aeruginosa	12	17.1
Escherichia coli	12	11.2
Salmonella enterica	12	17.2
Bacillus subtilis	12	6.6
Lactobacillus fermentum	12	9.1
Enterococcus faecalis	12	10.0
Listeria monocytogenes	12	10.2
Staphylococcus aureus	12	9.8
Saccharomyces cerevisiae	2	Not assessed
Cryptococcus neoformans	2	Not assessed

Table S5: Taxonomic classification of contigs co-harbouring two resistance genes

Sample	Farm†	Type‡	Gene groups	Class	Taxonomic classification*	Gene location on contig
DF0025	D1	FDE	*aph(3”), aph6*	Aminoglycosides	Proteobacteria	153 – 980<br>980 – 1721
DF0068	D4	FDE	*aph6, aph(3”)*	Aminoglycosides	Proteobacteria	95 – 931<br>931 – 1758
DF0097	D4	Soil	*emrRsm, emrCsm, emrAsm, emrBsm*	Drug and biocide	*Stenotrophomonas*	936 – 1379<br>1426 – 2869<br>2904 – 4061<br>4069 – 5636
DF0167	D1	WM	*crp,*<br>bla_{ACC}	Drug and biocide, β-lactams	*Enterobacterales*	21777 – 22405<br>113623 – 114795
DF0176	D1	FDE	*fosA*<br>bla_{PER}	Fosfomycin<br>β-lactams	*Gammaproteobacteria*	1499 – 2074<br>2148 – 3074
DF0188	D1	FDE	bla_{PER}<br>*fosA*	β-lactams, fosfomycin	*Gammaproteobacteria*	22 – 948<br>1022 – 1597
DF0188	D1	FDE	bla_{PER}<br>*fosA*	β-lactams, fosfomycin	*Gammaproteobacteria*	7225 – 8080<br>6576 – 7151

†D1, Dairy 1; D4, Dairy 4

‡FDE, Farm dairy effluent; WM, waste milk

*Classification recorded for the lowest taxonomic rank identified

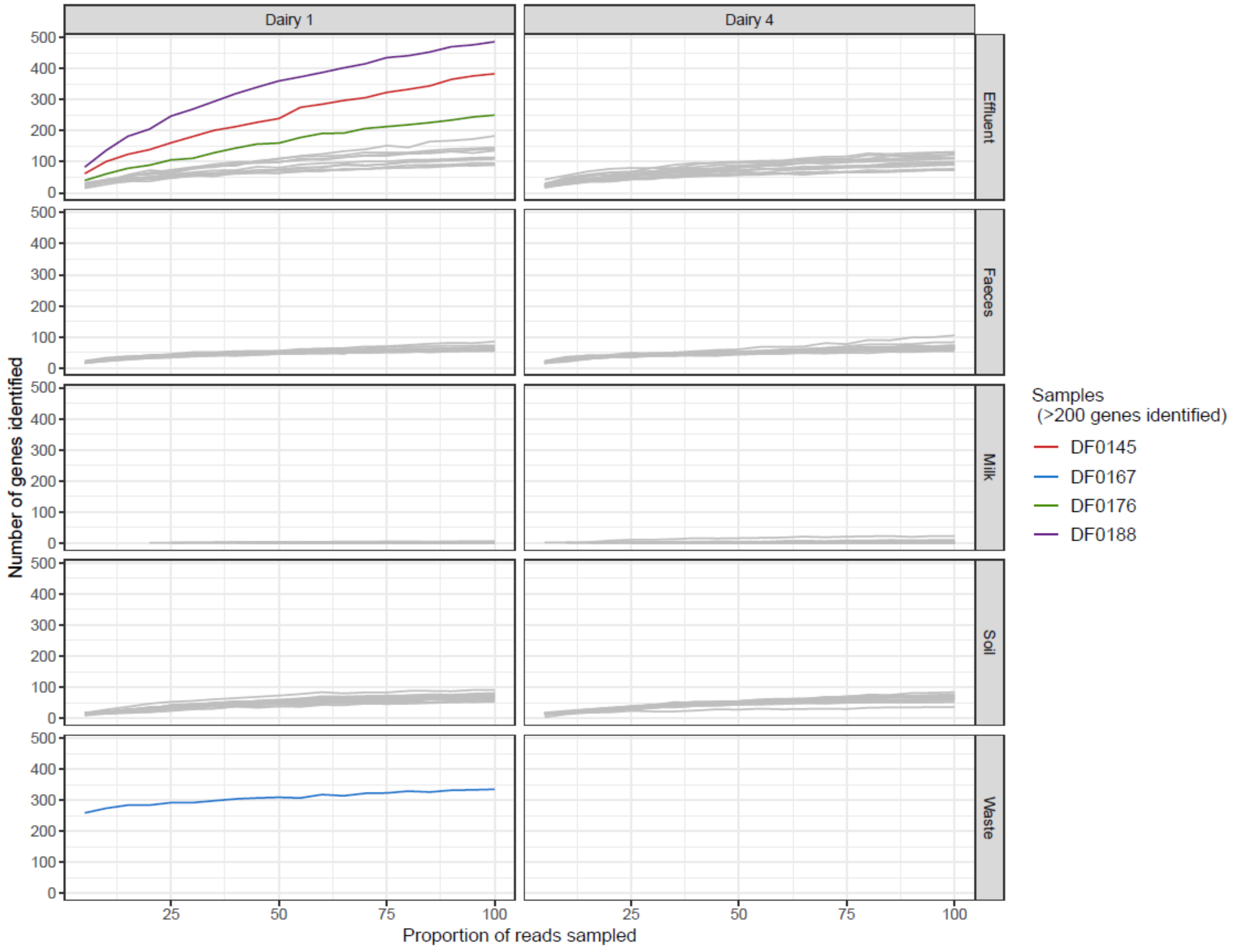

Figure S1: Rarefaction analysis of antimicrobial, heavy metal and biocide resistance genes detected in sequencing reads. Samples with > 200 genes detected are highlighted in colour as defined in the legend.

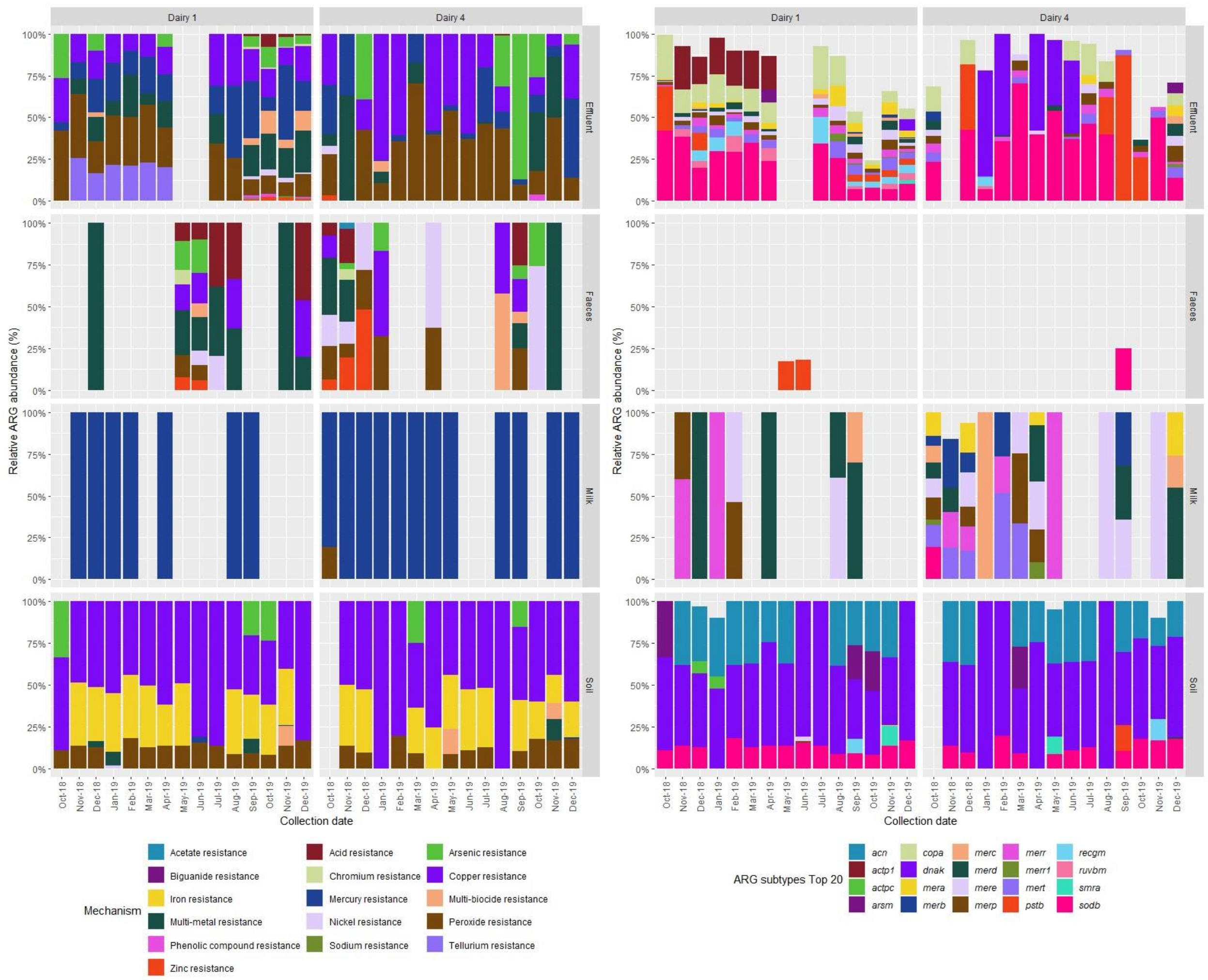

Figure S2: (A) Relative abundance of heavy metal and biocide resistance genes and (B) the top 20 most abundant heavy metal and biocide resistance genes identified from farm dairy effluent, faeces, milk, and soil samples. Resistance genes are classified at the class level.

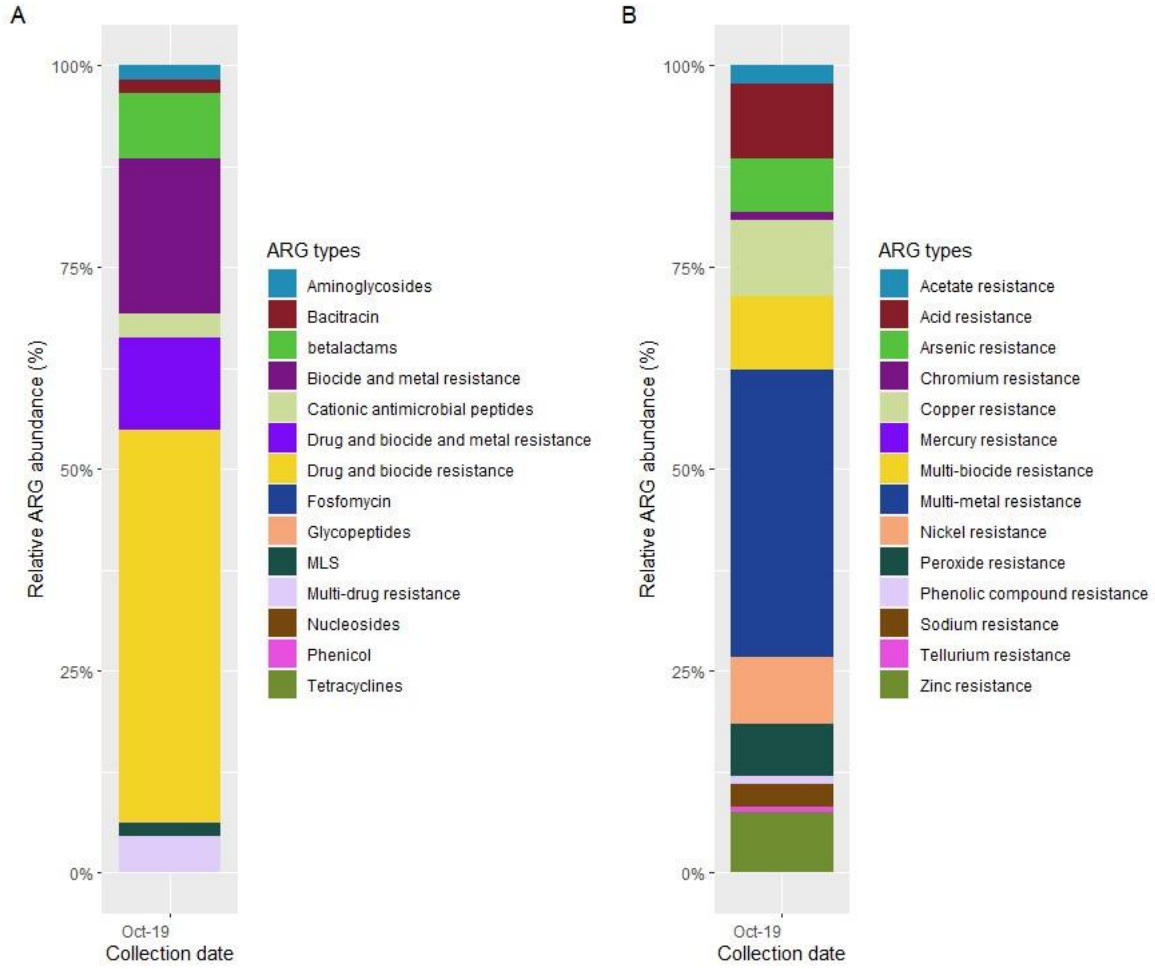

Figure S3: (A) Relative abundance of antimicrobial resistance genes and (B) heavy metal and biocide resistance genes identified from the waste milk sample (DF0167). Resistance genes are classified at the class level.

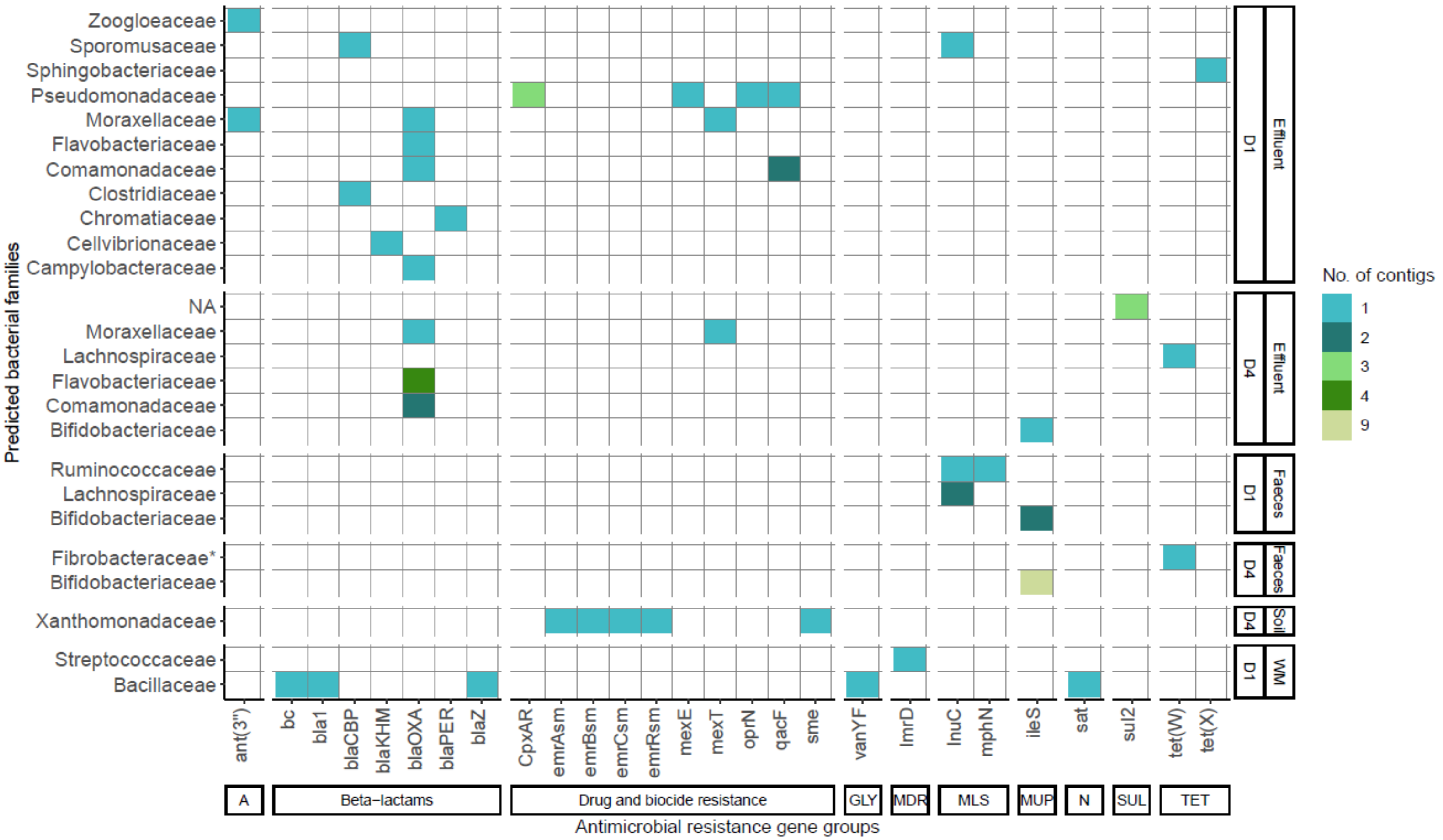

Figure S4: The predicted bacterial family of 58 contigs harbouring acquired antimicrobial resistance genes (ARGs). ARG groups were grouped per class of antibiotics and the contigs grouped by sample type and farm. The number of contigs with the unique family and ARG combination are indicated by the colour key on the Figure legend. A, Aminoglycoside; GLY, Glycopeptides; MDR, Multi-drug resistance; MLS, Macrolide, lincosamide and streptogramin; MUP, Mupirocin; N, Nucleosides; SUL, Sulfonamides; TET, Tetracycline; WM, Waste milk. The *Fibrobacteraceae* family is marked with an asterisk as this was the only family from the order *Fibrobacterales* that was present in the database. NA, Bacterial family not identified by contig classified as *Betaproteobacteria*.

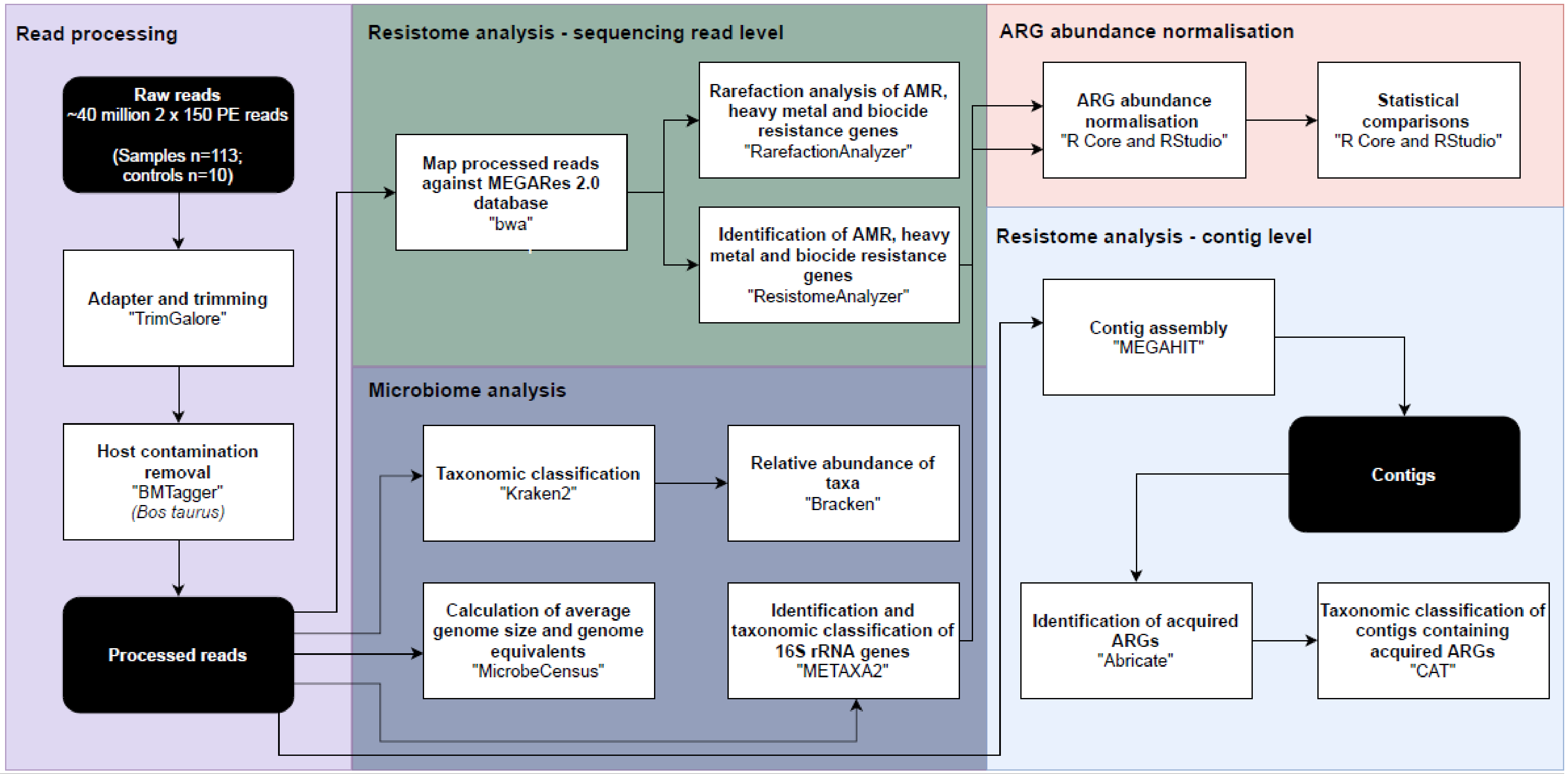

Figure S5: Workflow for bioinformatic analysis of shotgun metagenomic sequencing reads. Black rounded-edge rectangles stipulate the sample input for each analysis. White rectangles indicate the analysis steps and the tools used are shown in quotation marks. Arrows show the direction of analysis.
